# Spaceborne and spaceborn: Physiological aspects of pregnancy and birth during interplanetary flight

**DOI:** 10.1113/EP092290

**Published:** 2025-06-27

**Authors:** Arun V. Holden

**Affiliations:** ^1^ School of Biomedical Sciences University of Leeds Leeds UK

**Keywords:** birth, development, embryo, fetal, pregnancy, radiation damage

## Abstract

Crewed interplanetary return missions that are on the planning horizon will take years, more than enough time for initiation and completion of a pregnancy. Pregnancy is viewed as a sequence of processes – fertilization, blastocyst formation, implantation, gastrulation, placentation, organogenesis, gross morphogenesis, birth and neonatal development – each of which needs to be completed successfully, and each of which has a probability of success. The effects of the environment of interplanetary flight – microgravity and galactic cosmic rays (GCR) – on these probabilities are inferred from Earth and low Earth orbit experiments and observations and current models of morphogenesis. The principal hazards for intrauterine development are due to interactions with GCRs, where a variable flux of high energy particles would be interacting with a growing embryonic and fetal target volume, and produce linear tracks of ionization‐associated damage. Short term damage would be predominantly mediated via reactive oxygen species, and long‐term damage via DNA. Exposure to GCRs is expected to increase the probabilities of implantation failure and of premature labour. A live healthy birth would be possible, but its likelihood reduced. The long time scale of growth and development of the neonatal brain makes delayed manifestation of neurological or behavioural disorders likely.

## Abstract (highlights)

- **What is the topic of this review?**
  This review discusses the hazards posed by the galactic cosmic rays and µ‐gravity experienced in interplanetary space on cell, tissue and integrative human physiology during gestation, from conception to birth.
- **What advances does it highlight?**
  Physiological effects of cosmic ray tracks through tissue are not known, and will depend on how many, what sort and what energy. Embryonic and fetal growth increases the area for interactions with cosmic rays, and short‐term effects of cosmic radiation could interfere with developmental processes and increase embryo loss. Interactions with myometrial tissue, increasing its excitability and the likelihood of premature labour, would lead to increased fetal loss.

## 1 INTRODUCTION

Sexual activity and reproduction are a normal component of mammalian physiology, and reproduction in placental mammals is robust – they have evolved and survived on Earth over 225 million years, hominins 6–7 million years and humans for more than 200,000 years. Whatever the environmental constraints and stresses, human engagement with sexual activity has remained unfettered, and, in spite of low natural fecundity and contraceptive technologies, has contributed to continuing growth of the global population. The global population is predominantly urban, maintained by technologically based societies, and current technologies enable speculations about human exploration, discovery, exploitation and perhaps settlement of the moon and other planets.

Plans for prospective crewed missions to Mars and their return envision a small crew on a 2–3 year mission, with the outward and return journeys each of about 9 months. About 15% of space travellers have been women, and it is reasonable to assume that some of the crew on missions to Mars will be women. Women will be integral to any subsequent attempts at establishing permanent settlements. A return voyage to Mars would provide more than enough time for all the processes of human reproduction to occur. Such a contingency is outside any mission plans, but needs to be considered, to control its probability of occurrence, and to plan how to manage it should it occur. It would open opportunities for a unique case study, and could be viewed as an unplanned for, but acceptable mission byproduct. Just thinking about it identifies areas of reproductive physiology where mechanisms are poorly understood and research could be informative and perhaps useful in obstetric practice on Earth.

Pregnancy is not a straightforward deterministic process: both gestation and birth are hazardous and risky, and can be considered as stochastic and quantified by probabilities. Maximal fecundity, the rate of conceptions/menstrual cycle when trying to get pregnant, is about 25–30%, and only about a third of these conceptions end in a successful delivery. About 40–50% of conceptions terminate in clinically unrecognized early pregnancy loss, or in spontaneous early abortions (Norwitz et al., 2001; Wilcox et al., 1988). Some healthy couples fail to conceive for years; some pregnancies end in miscarriage or stillbirth, some births are premature, some babies are born with congenital defects. Weaning a heathy baby, a first step towards an independent and individual life, takes place after a series of necessary processes in a chain linking outcomes to the preceding outcome, and that all need to have been sufficiently successful. When there are data from a patient population the probabilities (Spiegelhalter, 2024) can be estimated from observed rates; when there is no data they can be inductively estimated a priori (Goodman & Salow, 2021) from models of the cellular, tissue, organ and integrative physiological mechanisms.

The risks can be quantified from the occurrence rates in large populations, as percentages: in the UK early clinical miscarriage occur in 10–20%, late miscarriage in 1–2% of pregnancies; still births in 0.4%, congenital heart defects in 0.7% and neural tube defects in 0.012% of births (Gallimore et al., 2024; Quenby et al., 2021).

The differences in precision reflect the different sample sizes and reliability of reported data. Hospital‐based statistics of births and still‐birth are reliable, of miscarriage less so, and statistics of conception and early embryo death rates are based on assumptions and limited data (Boklage, 1990; Jarvis, 2016). The statistics vary between populations, age and the availability of health care, and here data for young, fit, healthy women within an adequate health care system are used. The rates can be considered to be estimates of risks quantified by empirical conditional probabilities *P_i_*, 0 < *P_i_* ****< 1, in a chain linking outcomes to stages during gestation. *P_i_* is the probability of a successful outcome *i* given the previous outcome *i* − 1 had been successful, where success is a further step towards a live and healthy baby. The processes of pregnancy has been considered as a sequence of stages: the three clinical trimesters, the 23 Carnegie morphological stages of embryological development, the ∼40 cell cycles during embryonic and fetal development. Here pregnancy is considered in terms of 10 physiological stages – delivery of gametes, fertilization, blastocyst formation, implantation, gastrulation, placentation, organogenesis, gross morphogenesis, birth and postnatal development, each of which exemplifies different physiological mechanisms.

**FIGURE 1:**
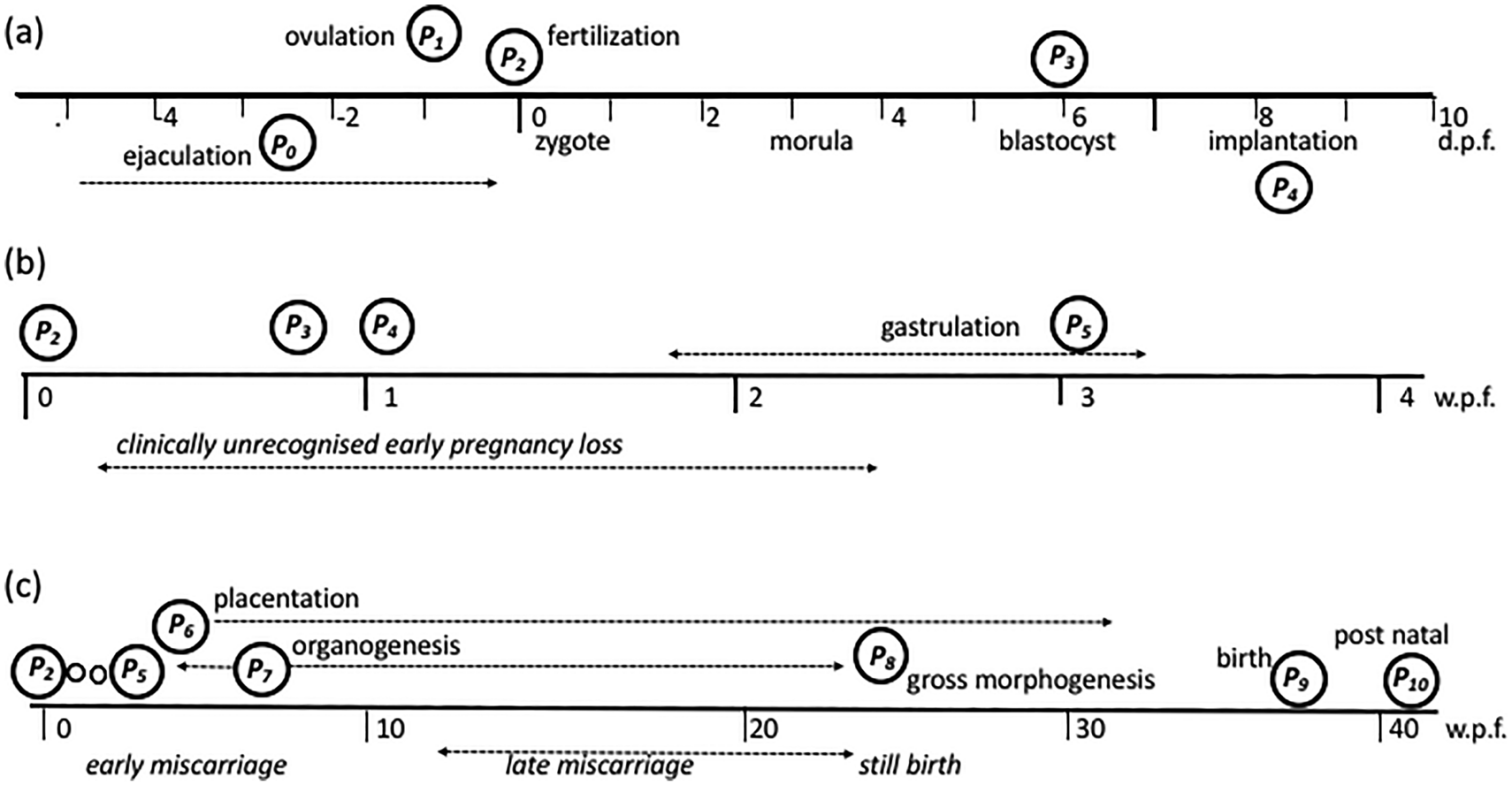
Timeline of development. *P*_0_ to *P*_10_ are probabilities of successfully completing the processes of ejaculation *P*_0_, ovulation *P*_1_, fertilization *P*_2_, blastocyst formation *P*_3_, implantation *P*_4_, gastrulation *P*_5_, placentation *P*_6_, organogenesis *P*_7_, gross morphogenesis *P*_8_, birth *P*_9_ and neonatal development *P*_10_, given the preceding event has occurred. (a) Pre‐fertilization to implantation, (b) stages in first 4 weeks of embryogenesis, (c) 40 weeks from fertilization to neo‐natal growth. The *P_i_* are located when the process is substantively complete, for example, for organogenesis all the organs are recognizably complete by 6 w.p.f., but not necessarily functionally competent until 25 WGA., when a premature baby is periviable (Patel et al., 2017). How each *P_i_*, is estimated, and its value, is discussed in the Appendix.

The sequence of physiological processes corresponding to the *P_i_* are mapped in Figure 1, and are not a strict temporal sequence: there can be overlap. *P*_0_ and *P*_1_, the delivery of gametes by active flagellar and passive motion, need to come together within a 24 h window for *P*_2_, fertilization, to occur. Figure 1a covers the time line from before fertilization to implantation; the timing of ejaculation is determined by behaviour, ovulation by the menstrual cycle, and in an individual can be imprecisely known. From fertilization to 2–5 days post‐fertilization (d.p.f.) precise data is available from in vitro fertilization studies, and to 14 d.p.f from applied reproductive technology (ART) studies. Figure 1b covers the first 4 weeks post‐fertilization (w.p.f.) to after gastrulation. In domestic practice, a woman might be aware she might be pregnant from 4–6 weeks’ gestation (WGA), but a pregnancy test for human chorionic gonadotropin levels (hCG > 10 µM) can be effective from 12 to 14 d.p.f. Gastrulation has begun before there is evidence of a pregnancy in vivo, and most fertilizations end in clinically unrecognized early pregnancy loss. Figure 1c covers the duration of a standard 38 w.p.f. pregnancy and early postnatal development. The processes of placentation, organogenesis and gross morphogenesis extend over time, and are located at the times when they are apparent. The evidential bases for estimating the values of the *P_i_* are outlined in the Appendix. The probability of delivering a healthy normal developing baby and it continuing development *P* is then the product *P* = *P*_0_ × *P*_1_ × *P*_2_ × *P*_3_ × *P*_4_ × *P*_5_ × *P*_6_ × *P*_7_ × *P*_8_****** × *P*_9_ × *P*_10_.

**FIGURE 2:**
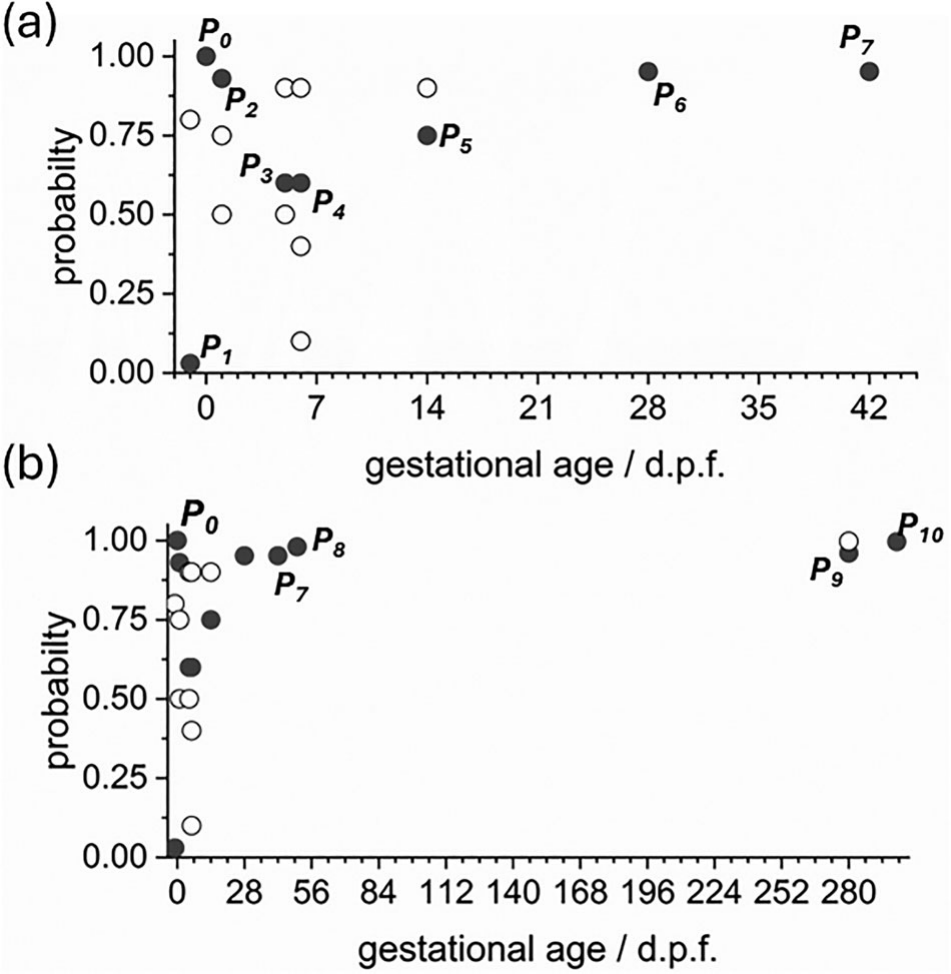
Transition probabilities *P_i_* of Figure 1 mapped onto gestational age. (a) Embryo from fertilization *P*_0_ to organogenesis *P*_7_. (b) Transition probabilities throughout gestation, into early postnatal development. The two values for *P*_9_ distinguish between born alive and born alive and well. Each data point is an estimate justified in the Appendix, and based on clinical observational studies and assumptions, and is a single fraction from a referenced study. Filled circles are values selected and used below, with their selection based on both the data source and compatibility with later survival probabilities.

Estimates of probabilities *P*_0_–*P*_10_ based on clinical research data are plotted in Figure 2a for the first 6 weeks of embryonic development, and Figure 2b for the entire period of gestation. The values and their sources are all given in the Appendix. Both panels start with an intravaginal sperm delivery event *P*_0 _= 1 within a day of ovulation as a given rather than speculate on what led up to it. Estimates of *P*_2_–*P*_4_ vary widely, as before a pregnancy is objectively identified by hCG level around 12–14 d.p.f. or by ultrasound around 6 weeks’ gestational age (WGA) its existence is uncertain, and estimates based on presumed ovulation times and of reported sexual activity are unreliable and inconsistent. Invasive embryo‐recovery procedures accompanying scheduled surgery 24 h after documented intercourse or from IVF studies have given precise but different probabilities of fertilization and implantation. Estimates for the fetal probabilities are precise and based on records of late miscarriage and stillbirth, and are consistently close to 1.

**FIGURE 3:**
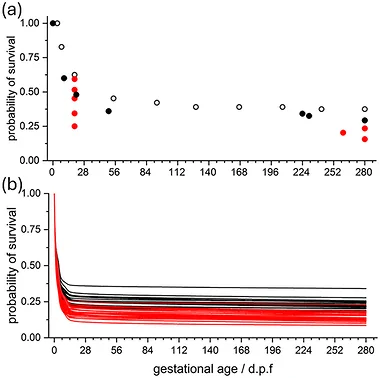
Probability of survival through embryo, fetus and birth following fertilization. (a) Open circles are from Boklage (1990), red circles from data from ART (blastocyst formation) and IVF ∼ full term births, filled circles model results computed from the products (*P*_1_ × *P*_2_ × *P*_3_ × *P*_4_.). (b) Probability of survival of embryo/fetus following fertilization with all *P_i_* independently stochastically depressed by 5% (black lines) and 10% (red lines).

Figure 3 plots the estimated and modelled probability *P* of the zygote surviving through embryo and fetus (Figure 3a) into babyhood throughout gestation, and (1 − *P*) is the cumulative probability of embryo and fetal loss. *P* is, by definition, monotone decreasing with time during gestation, and gives an overall *P* of a fertilization resulting in a live birth of *∼*0.3. Most of the 70% pregnancy loss is early, in the first trimester. Most of the early pregnancy loss occurs before implantation, and is clinically invisible and not noticed by the mother. Fetal survival after the first trimester is robust, with late miscarriage and stillbirth rates less than 2%.

The estimated survival probability in Figure 3a is from data in five longitudinal studies summarized in Boklage (1990) (open circles), and IVF studies (red). The computed survival probability (black circles) is from the black filled circle probabilities of Figure 2. Figure 3b illustrates modelled trajectories produced by decreasing all the *P_i_* randomly by 10% and 20%: fetal survival is robust, and depressed only into the range of IVF pregnancies.

Pregnancy and childbirth are part of normal human physiology, and are extended into social biology by the need for birth assistance. Pregnant women and their babies are resilient and can live in extreme conditions on Earth with simple clothing and sheltering technologies, at altitudes up to 4000 m and a temperature range between −30**°**C in the arctic and +40**°**C in the Kalarahi desert. They can also survive in the hostile environments produced by war and natural disasters. It may be easier and safer to complete a pregnancy within the safe engineered and confined environment of a spaceship in interplanetary flight than in many locations on Earth, for example, contemporary war zones.

Here some of the physiological consequences of initiating and completing a pregnancy far from Earth are considered, from the viewpoint of the embryo, fetus and neonate rather than of the health and life expectancy of the of adults. The effects of prolonged space flight on the longer term health and reproductive potential of adult astronauts after return to Earth is considered in Jain et al. (2023).

The aim is to consider how the *P_i_* might be changed if the pregnancy were to occur during interplanetary flight. The black swan hypothesis is that successful completion of a pregnancy during a prolonged space voyage is not possible: this could be falsified by a single birth *en route*.

First the hazards of interplanetary flight are considered, then the cellular, tissue and integrative physiology of the stages associated with *P*_0_ to *P*_10_ leading to live birth, and how the environment of interplanetary travel is expected to impact on them.

## 2 HAZARDS OF INTERPLANETARY FLIGHT

NASA's Human Research Program has itemized five hazards of interplanetary flight: (1) the hostile environment of space and (2) communication and logistics at large distances from Earth, both of which require engineering solutions; (3) the psychological effects of prolonged isolation require careful crew selection, management and motivation; and the physiological effects of (4) µ‐gravity and (5) radiation on the crew. Current spacecraft engineering technology provides a safe bubble of protection from the extreme vacuum and temperature of orbital space and beyond; here the effects on pregnancy of radiation, µ‐gravity and biological interactions within the restricted volume of the spacecraft are considered.

### 2.1 Solar and ionizing radiation

#### 2.1.1 Safety levels

The major source of information about the effects of ionizing radiation on humans has been epidemiological studies following survivors of nuclear incidents (Hiroshima, Nagasaki). In an atomic explosion there is an initial blast of neutrons and gamma rays, followed by long lasting exposure to alpha, beta and gamma radiations from the decay of radionucleotides in particulate fallout, which may be ingested or inhaled. Short term effects of intense exposure to ionizing radiation are deterministic – radiation sickness and death; long‐term effects are stochastic, with an increased risk of cancers and reduced life expectancy. Exposure safety limits that are physically based on gamma radiation do not simply translate to interplanetary particulate radiation, which is external and is of high velocity charged nuclei (and their interaction products). Exposure limits based on the risk of future cancers do not simply translate to embryonic and fetal developmental physiology and morphogenesis.

#### 2.1.2 Space scales

In classical physics radiation is modelled by particles or by electromagnetic waves. In the 1920s these alternative viewpoints were unified in quantum mechanics, which underpins much of modern technology and chemistry, but has had little impact on bioscience outside the sub‐nanometre to nanometre scales of the molecular biology of the energy transfers of oxidative phosphorylation and photo‐transduction (Marais et al., 2018). Quantum mechanics has been invoked to explain the unexplained, like navigation during bird migration (McFadden & Al‐Khalili, 2018) or consciousness (Hameroff & Penrose, 2014).

Ionizing radiation displaces orbiting electrons from atoms, and is usually described in terms of particles. The effects of ionizing radiation involve individual charged particles – atomic nuclei, with a size of 10^−15^ m – interacting with the intracellular environment at scales from 10^−12^ (atomic) through 10^−10^ m (molecular) to 10^−6^ m (organelle). The structure of this environment where molecular biology meets integrated intracellular processes has been sketched by electron microscopy (Heinrich et al., 2021). Within any one of the thousands of organelles, processes can involve moderate numbers of molecules – in a cell nucleus some two dozen DNA molecules, thousands of ion channels, 10^8^ histones – too many for modelling each molecule individually, and too few for continuous partial differential equation models.

On the organelle to cell scale, 10^−6^–10^−3^ m, the three‐dimensional visualization of intracellular structures and their dynamic interactions (for example, Sheard et al., 2019) is becoming sufficiently quantitative to be incorporated into computational models of sub‐cellular and cell physiology (Colman et al., 2017). The models are hybrid: continuous partial differential equations that interact with stochastic events.

On the cell‐to‐organism scale, 10^−6^–10^0^ m, physiological continuum models with variables and parameters that change smoothly in space and time have proved adequate.

#### 2.1.3 Solar radiation and wind

Interplanetary space in our solar system is bathed in sunlight, and showered with charged particles of the solar wind and galactic cosmic rays (GCR), with ionizing radiation levels more than two orders of magnitude greater than those on Earth.

The full electromagnetic spectrum of the sun ranges from ultraviolet (UV) to radio waves. UV can damage cells and tissues, with effects from sunburn to melanoma, acting directly on DNA leading to copying errors and mutations. UV can also act indirectly on DNA via reactive oxygen species (ROS) or free radicals (Agarwal et al., 2003; Schreier et al., 2015). The cellular and tissue effects of UV can be described in detail, since controlled in vitro biochemical, biophysical and physiological benchtop experiments on the mechanisms are straightforward to carry out.

The solar wind is composed of a magnetized plasma of protons and alpha particles (H and ^4^He nuclei) and electrons with energies of 1–10 keV; it is continually streaming outwards from the sun at 400–800 km s^−1^ and forms the heliosphere, a 2 × 10^12^ m bubble around the solar system. Solar activity is modulated by reversals in the sun's magnetic field on an ∼11‐year activity cycle, which is made manifest as the sunspot cycle. The solar activity cycle modulates the average GCR fluxes as the solar wind at the heliosphere boundary deflects some GCRs, to produce a 180° out‐of phase, 3‐ to 4‐fold modulation of interplanetary GCRs. On a time scale of months to years there are sporadic solar (energetic) particle events (SPEs) that last tens to hundreds of hours, with their probability of occurrence modulated in phase by the 11‐year solar activity cycle. In SPEs solar particles are accelerated by the magnetic fields of solar flares and coronal mass ejections. An SPE can produce a 10^2^‐ to10^3^‐fold increase in dose rate. Low energy solar particles with energies <30 MeV/*n*, where *n* is the number of nucleons (protons and neutrons) in the nucleus, are no problem as they would be blocked by the walls of the spacecraft, or even the material of a spacesuit.

#### 2.1.4 Galactic cosmic rays

Primary GCRs are stable, charged, high energy particles – electrons, and nuclei with atomic number *Z* from 1 (H) to 92 (U) – that originate from outside our solar system, and some of the heavier ions from outside our galaxy (Castelvecchi, 2017); occasionally they can have inexplicably high energies (Telescope Array Collaboration, 2023). Earth's magnetosphere and atmosphere shield life on Earth, and the magnetosphere shields voyagers in low Earth orbit (LEO) from GCRs. The horizontal component of the field shields Earth below from inbound GCRs, and the toroidal shape of the field gives 10‐fold lower GCR flux at the equator than the poles. Reversals in Earth's magnetic field have occurred at random intervals between 10^3^ and 5 × 10^6^ years. During these field reversals there were periods of reduced or zero dipole field when there would have been little or no magnetic shielding from primary GCRs. Secondary GCRs can be detected on Earth's surface as neutron fluxes and showers of secondary particles produced by interaction of GCRs with the atmosphere. These would have been increased by 15–60% at Earth's surface during a magnetic field reversal (Harrison, 1968).

Measurements of secondary GCRs from mountain observatories and high‐altitude balloons allow changes in primary GCR flux to be calculated using models. The first direct human subjective experience of GCRs was on the Apollo moon mission, as phosphenes, light flashes seen in the dark, believed to be ionization tracks in the aqueous humour of the eyeball produced by passage of single GCR particles (Narici, 2024). Direct measurement of GCRs is by interplanetary probes outside the protective magnetosphere of Earth (Boezio et al., 2020; Peng & Yuan, 2019).

Consider a sphere of interplanetary space large enough to encompass an astronaut – it is not empty, but bathed in radiation and bombarded by particles. During a period of time, a number of particles will pass through it: anisotropic GCRs from any direction and solar wind predominantly from the direction of the sun, with paths modified by solar and planetary magnetic fields. These primary particles can be characterized by how many (fluence, or rate of fluence, the flux), what sort (from^1^H^1+^ to ^238^U^92+^), and of what energy (eV, from <1 keV/*n* to over 10^5^ MeV/*n*, where *n* is the number of nucleons in the nucleus).

The GCR flux is of the order of 10^3^ atomic nuclei/s going through a m^2^ surface of a solid angle of one steradian at a few tens of GeV/*n*. As the atomic number *Z* increases from *Z* = 1 (solar and GCR protons) to *Z* = 34 the number of counts falls by three orders of magnitude (Durante & Cucinotta, 2011). This is ∼40 particles a year intersecting an area of 100 µm^2^ (a nominal cell), of which perhaps one particle has *Z* > 2.

GCRs contains all stable nuclei – any unstable nuclei will have disintegrated on their interstellar transit, but the relative abundance of the elements tends to decrease as *Z* is increased: by 65–75% for *Z* = 1 (protons/H), by 10–20% for *Z* = 2 (Helium), and by 10^−6^ as *Z* is increased to 26 (^56^Fe^26+^ iron ions). This decrease is non‐monotonic: for *Z* > 6 (carbon), the abundance of elements with odd *Z* is 10^1^–10^2^ greater than the abundance of their two even *Z* neighbours (Beatty et al., 2022). The abundance of GCR nuclei of *Z* = 1 to *Z* = 26 has been calculated from measurements and for 27 < *Z* < 92 from models of stellar and interstellar nucleogenesis.

The flux–energy spectrum, the number of particles (in GeV s m^2^ sr, from 10^3^ to 10^−27^) against kinetic energy (from 10^9^ to 10^21^ eV/*n*) shows a power law in energy, with an exponent of ∼3, over 32 orders of magnitude of flux and 12 orders of magnitude of energy. For kinetic energies <1 GeV/*n* the flux of GCRs is modulated 3‐ to 5‐fold by the 11‐year solar activity cycle, with high GCRs correlating with low solar activity.

If the sphere is in a spacecraft these characteristics will be modified by shielding by the material of the craft, and for particles with energies >50 MeV secondary charged particles and neutrons generated by spallation – nuclear interactions – of GCRs with this material. If the sphere is smaller and within a person, englobing an organ, tissue or cell, there will be in vivo interactions between the particles and the tissue producing additional cascades of secondary particles. The lighter secondary particles expand the volume of damage. Primary and secondary charged particles interact with covalent bonds and electron shells in the surrounding material, losing energy and producing ionization tracks. The energy transferred from a charged particle to the surrounding material/unit length of track is the linear energy transfer (LET) with units keV/µm.

As energy is transferred, the particle slows and stops, with a path length that is of the order of tens of centimetres in water for a 150 MeV/*n* proton or 1000 MeV/*n* ^56^Fe^26+^ iron ion, both plausible GCR particles. Taking water as a surrogate for tissue, GCRs can penetrate, pass through and transfer energy to tissue in vivo. The dose or absorption/kg of the organ or tissue is expressed in units of Gy (gray), where 1 Gy is equivalent to 1 joule of radiation energy absorbed/kg.

The absorbed dose is proportional to the product of LET and fluence, and depends on the composition of the absorbing material. The biological consequence depends on both the type of tissue and the type and mixture of particles, and multiplying the dose in Gy by an empirically determined quality/radiation weighting factor *Q* gives the biological risk or dose equivalent in Sv (sieverts),

The relative fluence in interplanetary space of 85% protons and 14% He^2+^ drops to <1% by *Z* = 3 and to ∼0.005% for *Z* = 15–25; while the dose/Gy falls from 50% for protons to ∼0.8% for *Z* = 15–25. The relative dose equivalent falls from 10% for protons to ∼5% for *Z* = 15–25 (see Figure 1 of Durante and Cucinotta, 2008). These values are computed and based on transport models of GCR formation and interactions (Ehresmann et al., 2016). GCRs would produce more ionization and biological damage via rare, low (<1%) fluence particles with *Z* > 3 than via the higher fluence (99%) *Z* < 3 particles of H^1^ and He^2^ nuclei.

The particle radiation environment of interplanetary space measured inside the Curiosity Rover in transit to Mars in 2011 showed during the 7 months of recording the average CGR dose rate was about 300 µGy/day, with proton fluxes of 0.22 cm^−2^ s^−1^ sr^−1^. For a nominal cell cross sectional area of ∼100 µm^2^ there would be a mean of ∼40 GCR tracks through a cell in a year, and ∼1 for particles with *Z* > 2. There were five SPEs during the 7 months, each with an ∼10‐fold increase in dose rate (Ehresmann et al., 2016; Zeitlin et al., 2013). Similar results have been obtained from other spacecraft (Rahmanian et al., 2025). The total radiation exposure was ∼95% due to GCRs and ∼5% due to SPEs. During intermittent SPEs any crew could avoid the large fluxes of energetic protons by hunkering down in a bunker with as much mass between them and the sun.

In terms of human voyagers, particles with energies <30 MeV can be ignored as they would be blocked by even the fabric of a spacesuit (Wilson et al., 2006). Primary particles, with *Z* > 4 and energy >50 MeV/*n*, and all secondary particles produced on board or in vivo form the GCR hazards.

The GCR particles and secondary particles resulting from their interactions impact on cells and tissue as linear tracks of ionization and excitation, centimetres long, with a dense core some nanometres in diameter surrounded by a penumbra, an up to ∼100 µm diameter cylinder of excited electrons, with a density that decreases inversely with the radial distance squared. This is radically different from the absorption pattern for X‐ or gamma rays, which is a diffuse, spatially homogeneous random scattering of absorptions. Linear tracks of radiation damage have been visualized in cell nuclei in human fibroblasts that have been exposed to high energy Fe ion nuclei and immuno‐stained for DNA breaks (Cucinotta et al., 2011). The different distributions of single‐ and double‐strand breaks, base damage and clusters of DNA damage produced by atomic nuclei in cell nuclei almost certainly will be expressed differentially, and the biological effects will not be the same as for the same dose/Gy of X‐rays. The effects of these tracks on cell and tissue physiology are experimentally accessible on Earth by bombarding experimental preparations with ion beams or multiple ion beams that simulate GCRs.

GCR simulators that can partially reproduce the particle spectrum of GCRs, and apply it to engineered dummies, small mammals in vivo, and cells or tissues in vitro, have been developed, with initial applications related to the mechanisms of tumorigenesis or neuro‐cognitive deficits (Huff et al., 2023). In multiple beams, the physical components (flux, spectrum, dose) of GCRs are linearly additive, while the biological effects can be non‐linear, with antagonism, synergy and saturation.

Medical effects of radiation exposure are considered in terms of risk to an individual, primarily as shortening of expected lifespan by cancer. Most national space agency exposure standards are for an upper limit of 50 mSv/year (Shavers et al., 2024): with a 3% increased risk of death (as a shortened life expectancy on return to Earth) being an acceptable level of risk for a mission. Since 2021 NASA has adopted a career exposure limit for both women and men of 600 mSv. With current propulsion and shielding engineering, these limits would be would be exceeded by the estimated 870 and 1200 mSv exposure for a return trip to Mars lasting 22–32 months (Simonsen et al., 2020). Even if all went well, after a safe return cancer risk would have been increased and life expectancy reduced.

Different tissues have different sensitivities to exposure to radiation – this is exemplified by acute radiation syndromes, with haemopoietic effects on bone marrow at 1–10 Gy, gastrointestinal effects at 6–10 Gy, and cardiovascular and CNS effects >50 Gy. This is consistent with an increased radiation sensitivity (Q‐factor) for tissue where there is active cell division and a short cell life. Developing and differentiating embryonic tissues, where there is cell division, differentiation and migration, will almost certainly have a higher radiation sensitivity than of fetal and postnatal tissue. These sensitivities could be quantitatively explored in pregnant small mammals, blastocysts and embryoids using simulated GCRs, using outcomes more informative than just cancer risk.

There have more than a hundred reversals in Earth's magnetic field during the deep time of placental mammalian evolution, during which there will have been increased GCRs on Earth's surface for periods lasting many generations: long‐term exposure to moderately increased levels of secondary GCRs has not prevented placental reproduction.

### 2.2 µ‐Gravity

On the surface of Earth the acceleration due to gravity, *g*, is ∼9.8 m s^−2^, and at the altitude of the International Space Station (ISS; ∼400 km) in low Earth orbit is about 0.89 × *g*. Satellites in orbit are in free fall – the only force determining their motion is gravitational. In interplanetary space the gravitational acceleration due to the sun and planets is <10^−6^ × *g*, micro‐enough to be mislabelled as zero‐gravity, but large enough to keep the planets in their paths.

Both electrical and gravitational interactions decay inversely with the square of distance and extend from sub‐atomic to cosmological space scales, but as a result of electro‐neutrality, gravity dominates at large scales. Size matters for an organism (Haldane, 1927) and for its physiology. In intracellular space the ionic and covalent bonds of intra‐molecular interactions and the hydrogen bonds, van der Waals, and induced dipole–dipole bonding of intermolecular complexes and interactions will dominate the bio‐ and physical chemistry that underpins cellular processes. In the millilitre to litre volumes of intercellular and intravascular fluid compartments, gravity will influence fluid distribution and pressures. Weightlessness will alter the forces on the musculoskeletal system.

After an initial disorientation, the acute effect of exposure to free fall in LEO is redistribution of fluid between compartments, and for prolonged exposures there are reduced exercise tolerance resulting from a reduced blood volume, pressure and heart rate; reduced muscle mass and strength; and increased bone loss. These adaptations are explicable in terms of reduced hydrostatic pressures and forces produced by the reduced gravity, and can be mimicked by prolonged bed rest. The centimetre scale, tissue level adaptations could lead to cellular adaptations, executed via reflex or other feedbacks. One would not expect changes in intracellular activity or structure to be directly produced by a change to free fall or µ‐gravity. Mammalian vestibular gravity receptors act by the distortion of sensory cilia by the inertia of fluid flow in active movements, or by the weight of otoliths in a gravitational field.

However, several weeks of spaceflight in LEO has been shown to alter gene expression in pathways associated with mitosis, apoptosis and differentiation in cultured human stem cells (Huang et al., 2025) and with cell proliferation and maturation in human induced Pluripotent Stem Cell (iPSC)‐derived neural organoids (Marotta et al., 2024). Gene expression was compared with that of ground controls. LEO provides a freefall µ‐gravity environment and exposure to GCRs, reduced 5‐fold by the magnetosphere and modified by interaction with the material world of the ISS.

An extensive proteomic and transcriptomic database from before, during and after long duration LEO and shorter higher orbit spaceflights shows similar transcriptomic changes, together with telomere elongation and changes in cytokines suggestive of stress (Overbey et al., 2024). The NASA twin study (Garrett‐Bakelman et al., 2019) followed a pair of monozygotic twin astronauts, one on Earth and the other on the ISS for a year and the year after return to Earth, with extensive monitoring of ‐omics, systems physiological variables and microbiome composition. Most of the changes and differences reversed after return to Earth. These before and after spaceflight studies provide extensive data that spaceflight results in cellular effects, but little mechanistic insights, and do not separate the effects of µ‐gravity, radiation, accelerations and stress of the leaving of, and return to, Earth. The effects of a simulated average µ‐gravity on fetal development could be studied on Earth in small pregnant mammals using rotating clinostats or random position devices

### 2.3 Closed Environment

Physiological experiments may be on isolated cells, tissues, organs, systems or individuals, but human physiology on Earth is not in isolation: an individual provides an ecosystem for hopefully few parasites, some commensalist mites, and an ∼10^13^ population of evolving microbials, and even more viruses and phages (Sender et al., 2016). Human physiology is not that of an individual in isolation; there is a necessary and continuous exchange of energy and matter between the ecosystem of their body and that of the environment. Current space stations – the ISS and Tiangong Space Stations – in LEO provide habitats of about 500 m^3^, need to be regularly resupplied, and maintain extensive microbial and fungal microbiomes (Urbaniak et al., 2022).

In the habitat of any manned mission to Mars, 1 kg of CO_2_ will be produced by each person per day, and removed by absorption and expulsion; O_2_ is replenished by electrolysis of water; and >98% of water can be recycled. The gases NO, H_2_S and CO are all endogenously produced, have physiological role as gasotransmitters (Guerra & Hurt, 2019; Oza & Kashfi, 2023; Prabhakar & Peers, 2014), and are present in  expired breath at 1–10 ppm (CO) and parts per billion. More than 2500 further volatile organic compounds have been identified by gas‐chromatography–mass spectroscopy with library matching from breath and bodily secretions and excretions that originate from physiological processes and the gut microbiome (Drabińska et al., 2021). No closed purification system is perfect, and over months the atmosphere of the spacecraft would become rank and rancid, and would need replenishing and refreshing.

Confinement of a small group of people for a long period of time, even if they share a common goal and are kept busy, can lead to strains in interpersonal relations, and psychological issues, anecdotally known as cabin fever. There have been several psychosocial isolation experiments aimed at simulating a trip to Mars that suggest mental health will be as important as physical wellbeing (Basner et al., 2013, 2014). Robust mental health could be achieved by careful crew selection, mission planning and monitoring, but tightly knit family groups have proved resilient and effective, as in the successful prehistoric colonization of the world outside Africa.

## 3 BECOMING PREGNANT AND FERTILIZATION: *P*_0_, *P*_1_, *P*_2_, SPACE SCALE <100 µm

Being pregnant in space means becoming pregnant must have occurred, before departure on Earth, a lunar base or orbit, or after departure in interplanetary space. Leaving the gravity wells of Earth or moon, or of orbits around them during an early undetected or unreported pregnancy would lead to exposure to accelerations of about 3 × *g*, much less than the sudden deceleration that triggers an airbag in a car, and of no consequence.

### 3.1 Coitus

Foreplay and penile penetration in freefall and µ‐gravity will require manoeuvres similar to those for making out and having sex while out of depth and free floating in the sea. No one is on top, any push leads to separation, and there is a need to hold on, and to control relative rotations. A hammock, not a bed, would be a useful adjunct. The vasculogenic hydraulics of sexual arousal and increased secretory activity result from increased flow in small arteries determined by their diameter, controlled by vascular smooth muscle and capillary pericytes (Davis & Atwell, 2023) and fibroblasts (Guimaraes et al., 2024).

µ‐Gravity should not inhibit erectile function or ejaculation; this is consistent with anecdotal reports from astronauts and cosmonauts who have experienced prolonged stays in orbit.

On ejaculation a few millilitres of semen, with 100 million sperm/mL, is deposited as a viscous glob into the moist vaginal head, about 15 cm from any ovum that is *en route* to, or within, one of the fallopian tubes. Migration of sperm is by active swimming in a viscous, inhomogeneous complex fluid (Hunter et al., 2011). The free‐floating ovum is passively drawn into the fallopian tube and wafted along by rhythmic synchronized beating of epithelial cell cilia lining the fallopian tube. Of the 10^8^–10^9^ sperm, about 10^6^ enter the uterus and 10^1^–10^3^ reach the vicinity of the ovum; fertilization is by one sperm.

For a sperm to deliver its tightly packed payload of haploid DNA it requires a source of fuel, a motor, and control over direction: these are all aspects of molecular and cellular physiology. Sperm motion is determined by the microfluid dynamics and continuum mechanics (Gaffney et al., 2011) of the heterogeneous media it is travelling through. The fuel is ATP and the motor is a system of microtubules (the axoneme) containing dynein motor ATPases (Inaba & Shiba, 2018). The internal anatomies of the cervix, uterine endothelium and fallopian tubes act as boundary conditions that guide the direction of travel.

Sperm need to spend time in the female reproductive track, or in assisted reproduction technology (ART) studies in an appropriate chemically defined medium composition, before they are capable of fertilization. During their travel the sperm undergo cellular processes of capacitation (Puga Molina et al., 2018) and hyperactivation (Tufoni et al., 2024; Zaferani et al., 2021) before they are capable of fertilization.

### 3.2 Sperm swimming

Even at the best of times, 40% of the sperm may not be structurally normal and well behaved, and fail to exhibit a progressive motion. Sperm can swim progressively in an aqueous medium at about 10–100 µm s^−1^, and have about 15 cm as the crow flies to swim from the cervix to the oocyte at the ampulla of a fallopian tube. Sperm has been recovered in fallopian tubes that were surgically excised 10 min after intravaginal insemination (Suarez & Pacey, 2006), and rapid transport of sperm through the uterus may be aided by travelling waves of myometrial contraction that have been visualized by cine MRI (Togashi, 2007) and ultrasonography (de Vries et al., 1990).

With regard to guided motion of sperm, the elongated geometry of sperm means that even thermal Brownian motion is anisotropic and progressive (Fakhri et al., 2010). Sperm swim at the same speeds in saline or semen or mucus, in spite of a 100‐fold change in drag due to the different viscosities. They swim further in a straight line in mucus than in saline, perhaps guided by threads of the cervical mucus (Katz et al., 1978).

With regard to collective motion of sperm, even in saline sperm engage in synchronized swimming, in which they mutually orientate against the flow, with flagellar phase and frequency entrainment due to hydrodynamic coupling. At the boundary between low viscosity (a few pascal.second (Pa.s) and high viscosity (∼100 Pa.s) media, sperm gang up, head to head, forming collective groups. These collectives move in the high viscosity medium at a higher velocity than could be achieved by single sperm. This cooperative behaviour is only exhibited by sperm with high DNA integrity, and is decreased by capacitation (Xiao et al., 2023).

All these mechanisms of sperm motion involve fluid mechanics – movement of the sperm in a moving medium, where the complex interplay of forces and geometry can be integrated into computational fluid dynamics models (Diemer et al., 2021) or simplified by considering just the spatial scales (Gaffney et al., 2021). The dimensionless Reynolds number Re for an object moving in fluid is the ratio between inertial and viscous forces. Re for a human sperm in saline is low, ∼0.1, and so inertial forces are negligible compared to hydrodynamic forces. The insensitivity of the progressive swimming velocity of sperm to the viscosity of the medium does not follow from fluid dynamics, but from the pattern of flagellar movement – the beat pattern – and the power output from the dynein motor (Gaffney et al., 2021; Ishimoto et al., 2018) is changed by the viscosity. The flagella beat pattern also influences the direction of progressive swimming (Gong et al., 2020).

### 3.3 Fertilization

The intimate approach of a few thousand fit and fertile sperm to a fresh oocyte does not guarantee fertilization; hence the use of intra‐cytoplasmic sperm injection in IVF. As the sperm binds to the zona pellucida, the acrosome releases enzymes allowing proteins on the sperm and egg membranes to bind and their membranes to fuse – these are membrane–protein and membrane–membrane interactions (Primakoff & Myles, 2002). Fertilization triggers intracellular calcium oscillations and waves that propagate by calcium‐induced calcium release at a velocity of 5–50 µm s^−1^ (Leybaert & Sanderson, 2012), that is, involving non‐linear wave behaviour in an excitable medium (Holden et al., 1991).

#### 3.3.1 µ‐Gravity

The low Reynolds number and length scale of a sperm implies sperm motion – the velocity of free swimming sperm and the effects of boundaries is expected to be the same in µ‐gravity and orbital free‐fall as on Earth. One would not anticipate problems in fertilization, but cohabiting rats and mice have been reported to fail to develop pregnancies in LEO. Fertilized mice are fickle, and readily resorb their embryos (Hofmann et al., 1987).

#### 3.3.2 GCRs

The sperm and oocyte haploid nuclei that would form a zygote contain chromatin as highly condensed protamine–DNA toroids (Moritz & Hammond, 2022; Ribas‐Maynou et al., 2022), together with RNA, proteins and perhaps centrioles, and some of their DNA is methylated, all of which may later regulate embryo progression and gene expression. The chromatin with ∼1 m of tightly packed linear DNA strands is the site for mutagenic interaction with radiation, but the sperm nucleus (2–3 by 3–5 µm and oocyte nucleus (about 10 µm diameter spheroid) are small targets, with cross sectional areas of ∼10–100 pm^2^. They are transient targets for CGRs; the average sperm lifetime is <50 days (Teves & Roldan, 2022), the oocyte is exposed to GCR from the time of leaving Earth's protective magnetosphere until the day after its ovulation, when it may be fertilized. For any oocyte and sperm that actually end up in fertilization, the ovum haploid nucleus will have had a 10‐fold greater probability of GCR interaction because of its size, and after 50 days of spaceflight the egg will have a further continually increasing probability of GCR interaction until its ovulation. GCR mutations in maternal genes should be a >10‐fold more likely than in paternal genes – this is counter to the paternal prevalence of human germline mutations (Gao et al., 2019).

However, any pre‐fertilization close encounters with GCRs would interfere with mitochondrial and cytoplasmic (acrosomal and flagellar in the sperm) function (Teves & Roldan, 2022), and could prevent fertilization. Given the GCR proton fluence of ∼0.2 cm^−2^ s^−1^ sr^−1^ the mean rate of GCR interactions with the to‐be‐chosen gamete preceding its participation in fertilization will be ∼10^−7^ s^−1^, or a Poisson process with a mean rate of 1 every ∼100 days. During the 24 h when the oocyte is available for fertilization, and if viable sperm are present, the probability of a GCR interaction with either of the haploid gamete nuclei that makes it is <1%.

In the adult testis spermatogenesis is ongoing, producing about 1–3 × 10^8^/day, and GCRs could impact on diploid spermatogonia, leading to cohorts of mutated or morphologically or physiologically abnormal sperm. Any accumulative effects of GCR impact on sperm count and mobility could easily be manually monitored during interplanetary flights.

## 4 DEVELOPMENT OF BLASTOCYST, GASTRULATION AND IMPLANTATION: *P*_3_, *P*_4_, *P*_5_, SPACE SCALE 100 mm TO MILLIMETRES

During the first week post‐fertilization, the fertilized zygote is moved by peristalsis within the fallopian tubes, and wafted by ciliary beating towards the uterus, while undergoing repeated mitoses, doubling cell number about every day.

### 4.1 First mitosis

ART studies show fertilization to be highly error prone; these errors could contribute to the preclinical pregnancy loss of *P*_3_ and *P*_4_, and early pregnancy loss of *P*_5_ to *P*_8_. Only half of in vitro fertilized oocytes develop into blastocysts, and, of those that do, many have aneuploidies that lead to pre‐ or post‐implantation developmental failure (Gruhn et al., 2019).

Chromosome dynamics have been visualized in vitro using fluorescent labelling, together with the behaviour of IVF embryos at the equivalent post‐fertilization times, and clinical outcomes of IVF implanted embryos obtained from the same donors that had been fertilized and nurtured under the same protocols (Currie et al., 2022). The first mitosis is long drawn out and inept, showing micronuclei characteristic of segregation errors in a third of research embryos and in a quarter of the embryos visually rejected for implantation. Some of the visible signs characteristic of post‐fertilization errors were seen in IVF embryos that were then implanted and led to live births, so the errors in a quarter to a third of the first mitoses can be non‐lethal and may be later corrected in vivo, presumably by excluding aneuploid cells in further development.

In the first 24 h after fertilization, there is intracellular unpacking, repacking and re‐arrangement, brought about by choreographed intracellular flows and transport. The cortical paternal and eccentric maternal pronuclei are moved towards the cell centre, giving the zygote more spherical symmetry than the oocyte, the chromosome sets brought together, centrosomes formed, and the maternal meiotic spindle is replaced by the developing mitotic spindle (Coticchio et al., 2023). A feltwork of cross‐linked actin filaments beneath the cell membrane tightened by membrane‐attached myosin motors procures the cell rounding and cleavage of the mitotic division of the zygote into two cells. The first mitosis can lead to euploidy and subsequent reduced implantation rates: many zygotes fail to reach first base of a successful mitosis into a two‐celled embryo, but if only one of the daughter cells is wholesome, a viable fetus and embryo can still emerge.

### 4.2 Cleavage cascade to morula

In subsequent cleavages cell integrity is maintained by membrane tension and osmotic pressure, and adhesive coupling to neighbouring cells. Defects from the four‐ to eight‐cell stage onwards can prevent development to blastocyst. During the next six cleavages the cells get smaller while the embryo remains ∼100 µm in diameter. The cells have low biosynthetic rates that are maintained by diffusive transport: cleavage is repackaging, rather than growth. Compaction into a denser inner cell mass, produced by actin–myosin interactions in the cell cortex and increased intercellular adhesion begins in the 4 d.p.f. embryo, in which cell–cell contact area is increased and their surfaces exposed to the outside medium reduced (Firmin et al., 2024).

The overall structure is still spheroid, and can be idealized as having the symmetries of a sphere, with no poles or faces. In the compacted cell mass, cells on the outside begin to differ from cells on the inside. Internalization can be explained by simple mathematical combinatoric and geometric (Shipley et al., 2009), or physical (Cockerell et al., 2023) arguments: it is not just a consequence of having more and more cells in the same volume, with some happening to be on the inside and some on the outside. Cells that form the surface of the morula begin apico‐basal polarization. There are also orientated cell divisions at the surface, so the daughter cells are stacked outside‐in, and cell sorting by surface cells with higher surface tensions (denser actin–myosin interactions in the cell cortex) pulling themselves into the interior. The cells are pluripotent, and their position determines their fate (Maitre, 2017) – the inner cell mass will develop into the embryo body and yolk sac, and the outer cells form the tropectoderm that will invades the maternal endometrial tissue during implantation (Firmin & Maitre, 2021; Firmin et al., 2024) and form the placenta.

### 4.3 Blastocyst

Fluid flow following Na^+^ pumping through the polarized surface of the trophoblast produces the expanding blastocoel cavity that inflates the embryo and pushes the inner cell mass to one side to form the embryo pole. The blastocyst has undergone a 10‐fold inflation in volume and a symmetry breaking as hydrostatic pressure in the blastocoel pushes and flattens the trophoectoderm epithelium.

Up to the peri‐implantation stage data are from ART and observations during IVF, and the behaviours are similar to those seen in murine early embryogenesis, where the mechanisms have been investigated experimentally by genetic, genomic and molecular biological techniques (Dard et al., 2008; Molè et al., 2020; Munisha & Schiment, 2021; Wang & Dey, 2006).

Human embryos have been maintained up to 13 days, and self‐organize a bilaminar disc, a pro‐amniotic cavity, a prospective yolk sac and differentiated trophoblast (Deglincerti et al., 2016; Shahbazi et al., 2016; Torre et al., 2023). Organoid models of the blastocyst have been developed, in which the trophectoderm and inner cell cluster lineages of the blastoid are expressed in the same sequence as seen in IVF human blastocysts (Kagawa et al., 2022). Blastoids produced from human embryonic stem cells can attach in vitro, self‐organize and form gastruloids (De Santis et al., 2024). Implantation begins in vivo at 7–8 d.p.f. as the trophectoderm attaches to, and penetrates through, the epithelial layer into the endometrial stroma (Ruane et al., 2022).

### 4.4 Implantation

From about 4 d.p.f., absorption of uterine fluid causes closure of the lumen of the uterus, placing the ∼100 µm diameter blastocyst in close contact with the endometrial epithelium. A positioning via interdigitating microvilli enables intercellular adhesion and the invasive cellular migration of implantation.

Before implantation all cells in the embryo are within 50 µm of the uterine luminal fluid bathing the blastocyst and O_2_, molecular signals and metabolites are exchanged by diffusion. Implantation requires both a receptive endometrium (Zhang et al., 2013), resulting from ovarian oestrogen and progesterone and locally produced signalling molecules, as well as a competent blastocyst (Norwitz et al., 2001; Cha et al., 2012). Implantation and placentation are accompanied by the development of the embryonic vascular system. This angiogenesis allows growth beyond the 100 µm constraint imposed by diffusion; the capillaries exchanging metabolites with cells are all ∼100 µm from all cells.

The processes of implantation in the human at 7–10 d.f.p. – apposition, adhesion, and invasion – cannot be studied in vivo or in vitro. These early stages of differentiation all use the same intracellular mechanisms of filament (actin, tubules) – motor (myosin, dynin) interactions, trans‐membrane transport that produces pressure gradients and fluid flows, and intercellular adhesive interactions and cell migration that are found in later growth, development and remodelling. They are all on the space scale of micrometres to millimetres. There is an exponential increase in cell number, to ∼2^10^, and so an apparent shift from discrete events to smooth processes.

### 4.5 Gastrulation

In preserved embryo specimens, gastrulation is defined as starting with the visible appearance of the primitive streak at 14 d.p.f., and ending by 19–21 d.p.f., when the oval embryonic disc is 1.5–2.5 mm long, and the embryo is a three layered structure with a midline and anterior–posterior axis, and neural groove, neural fold and emerging somites can be identified.

Gastrulation has been approached using transcriptomic and spatial mapping analysis of single ∼16–19 d.p.f. and Carnegie Stage CS 7 fortuitously acquired embryos (Cui et al., 2025; Tyser et al., 2021) to map the cellular processes in gastrulation, rather than just the appearance of its morphological features. Cell population, RNA velocity vectors and diffusion pseudo‐time plots suggest cell movement through a bifurcation from epiblast into mesoderm and ectoderm via the primitive streak. The 100 µm to millimetre scale morphogenetic changes during gastrulation could be imagined as continuous deformations of plastic layers, but are produced by movements of individual cells – migration, divisions and shape changes – produced by actin–myosin ratcheting of the intracellular cytoskeleton. The movements are constrained by cell–cell adhesion, controlled by chemical signals diffusing in an antero‐posterior, dorso‐ventral coordinate plane, and choreographed in time. As an ∼2D tissue grows it inflates, the intrinsic distance between any two cells increases. Curving in 3D can reduce the strain and energy required to maintain tissue structure, but leads to incompatibilities at ∼1D edges that can be resolved by the formation of cusps. Some of the morphogenetic changes during gastrulation – the development of grooves and folds – may be robust as they result from geometrical and physical constraints rather than biological programming (Collinet & Lecuit, 2021).

#### 4.5.1 µ‐Gravity

At the 10 µm to millimetre space scale of fertilization and implantation, the forces due to gravity are all negligible compared to the intermolecular and surface forces. in vivo, after fertilization, the 100+ cell, 100–200 µm diameter human blastocyst is free floating within fluid lining the uterine cavity. In the second week post‐fertilization the trophoblast adheres and implants into the endometrial mucosa. Throughout these 2 weeks, forces acting on the blastocyst are intermolecular – cohesive and adhesive – or produced by interactions between actin and myosin‐like filamentous proteins, or visco‐elastic or fluid flows. One would not expect µ‐gravity to affect blastocyst formation and gastrulation.

Two‐cell frozen mouse embryos have been frozen, transported to the ISS, thawed and successfully cultured into blastocysts in the freefall and radiation environment of the ISS (Wakayama et al., 2023). However, although blastocyst formation in mice is possible in LEO, in simulated µ‐gravity on Earth using a 3D clinostat, blastocyst formation had a lower growth rate, with fewer trophectoderm cells, but with normal polarization, and gave rise to fewer live births when the blastocysts were implanted (Wakayama et al., 2009). The birth rates for µ‐gravity cultured embryos was16% compared to 37% for 1 × *g* cultured controls: this reduction in the probability of survival falls within the range of trajectories of Figure 3b.

The <10 min of 3 × *g* acceleration during lift off could produce intra‐abdominal fluid flow on a centimetre to tens of centimetres space scale that could lead to an increased probability of an ectopic pregnancy if fertilization had occurred on Earth in the night before liftoff.

#### 4.5.2 GCRs

The biochemical processes during cell cleavages, blastocyst formation and cell movements and their local control would all be denatured by the energy deposited on intersection with the ∼10 nm shaft and scrambled by >100 µm penumbra of a single >100 MeV/*n* GCR ionization track. Such a locally catastrophic impact would be invisible and unremarked as pregnancy would not have been detected.

More than a thousand mouse two cell embryos have been successfully cultured into blastocysts in an automated incubator on the Shi Jian‐10 recoverable satellite in LEO exposed to ∼0.15 mGy/day (Lei et al., 2020). All stages from cleavage to blastocoel expansion were observed, but the percentage of embryos that developed into blastocysts (34% compared to 57%), and the average number of cells (41.5 compared to 53.9) in the blastocysts was less than controls cultured in an identical incubator on Earth. Orbital flight reduced the probability of blastocyst formation from 0.57 (close to the estimate of 0.6 for human *P*_3_) to 0.34. Following fixation and return to Earth, immunostaining of markers for trophoectoderm, inner cell mass, ectoderm and primitive endoderm showed differentiation had occurred, but was compromised, both semi‐quantitatively and in terms of the spatial distribution of the cells within the embryo. Markers for double and single strand DNA repair were higher in the blastocysts cultured in space, indicative of DNA damage. DNA methylation profiling showed a lower cytosine–guanine methylation, especially in differentially methylated regions related to histone, histone H4 acetylation and chromosome organization. High methylation regions were related to embryonic development, regulation of RNA, metabolic processes and regulation of intracellular protein transport.

These changes were mimicked in experiments on Earth with exposure to 0 5–2 mGy gamma radiation from ^137^Cs^55^, but not by simulated µ‐gravity. Blastocysts exposed to gamma radiation on Earth and implanted into pseudo‐pregnant mice could lead to live births, and so could be competent. Live birth rates were reduced from 34.6% (similar to that for IVF pregnancies in humans) to 7.45% for 2 mGy and 21.07 for 0.5 mGy.

Spontaneous monozygotic twins occur in 0.4% of live births, and fusion chimaeras have been occasionally identified: these result from preimplantation irregularities, and GCR exposure might be expected to make them more likely.

## 5 EMBRYO ORGANOGENESIS AND PLACENTAL DEVELOPMENT: *P*_5_, *P*_6_, SPACE SCALE MILLIMETRES TO TENS OF CENTIMETRES

Organogenesis starts with the embryonic heart at 21 d.p.f. as the conceptus reaches about a 500 µm radius and an embryo circulatory system and functional placental exchanges between maternal and embryo circulatory systems allow rapid growth in embryo mass and volume to occur (Abduljalil et al., 2012, 2019). In terms of structures, it makes sense to follow the development of different organ systems, and how they integrate with the embryo and with maternal systems. In terms of function, considering the heart, vascular system and placenta as separate entities misses their physiological role of perfusing the developing microcirculatory system.

### 5.1 Organogenesis

Single cell and transcriptomic data from *ex vivo* embryos are being applied to map development of different human tissues and organs (Zeng et al., 2023), and in principle can be mapped into the 3D reconstructions of the embryo, and its graduation into a fetus at 6 w.p.f. Most of what is known about human embryo development after 14 d.p.f. is from histological examination of archival material, or occasional *ex vivo* samples, and is predominantly descriptive morphology. Human early embryos are infrequently available for research, and specimens in historical collections (Yamaguchi & Yamada, 2018) have been re‐imaged by both MRI and histological methods, digitized (Dhanantwari et al., 2009) and 3‐D reconstructions made (de Bakker et al., 2016).

Given the intricate detail of the morphological developments between the three layers of gastrula at 18 d.p.f. and the fetus at 6 w.p.f. with all organ systems sufficiently developed to be recognizable, this section focuses on the heart. Analogous affairs of cellular differentiation, growth, division and migration; tissue formation from bringing together flows of migrating cells from different locations and of different types, and by local differentiation; vascularization; organ and body morphogenesis occur in all other organs and systems, even though the organs may not be viably functional until 22–24 w.p.f. The tissue processes can persist into adult life as functional or pathological remodelling.

Non‐invasive magnetic resonance imaging (MRI) provides millimetre scale resolution in vivo of organ and tissue structure, in clinical imaging and clinical and physiological research. MRI has been used to visualize the beginnings and ends of pregnancy (Schultz et al., 1999) and birth (Ami et al., 2019; Bamberg et al., 2012) but, because of safety concerns (de Wilde et al., 2005), not in longitudinal studies on what happens in between.

The first functioning embryo organ, the heart, begins rhythmic beating between 20 and 30 d.p.f. This has been detected by ultrasonography in IVF‐induced pregnancies, with known day of fertilization, and is consistent with data from natural fertilizations, where day of fertilization is estimated (Männer, 2022).

Mid‐gastrulation heart fields develop in the mesoderm, and the differentiation of cardiomyocytes starts within the first heart field. These myocytes migrate and will end up in the ventricular wall. The heart becomes identifiable at ∼26 d.p.f. as vascular networks develop into the heart tube, and by 28 d.p.f. begins beating (Buijtendijk et al., 2020; Hikspoors et al., 2022). The formation of a functioning organ requires the bringing together of cells of different types and functionality – capillary epithelial cells, fibroblasts and myocytes – and the construction of extracellular matrix, and linking in with other systems via the developing vascular system. Cells can migrate individually or *en masse*, as flows. These have been tracked using single cell RNAseq in 9–16 w.p.f. human hearts (Farah et al., 2024). While these processes of differentiation and morphogenic movements are carrying on, the embryonic systems and the embryo itself need to remain viable and functional.

Tissue architecture – the arrangement of different cell types around their perfusing capillaries – can be mapped and quantified histologically. Molecular mapping by single‐cell transcriptomics has led to the identification and quantification of new types and subtypes of cells: Litviňuková et al. (2020) present a detailed atlas of the adult healthy heart, comprising 11 major cell types, with the cell types composed of differentially distributed subpopulations. Organogenesis involves not just cell migration and growth, but also the integration of the movement of the multiple streams of different cell types, all while maintaining local functionality and embryonic viability.

The development of myocardium can be studied using non‐destructive diffusion tensor MRI of *ex vivo* examples, and the architecture can be quantified by the organization of the orientation of the myocytes. This is physiologically meaningful, as cardiomyocyte orientation determines the direction of local propagation, and of local force development. With 4.7 T MRI, Pervolaraki et al. (2013, 2017) have mapped the increasing organization of the ventricular myocardium from 93 to 143 days’ gestational age (DGA), as the developing ventricular myocardium acquires its mature compact (Jensen et al., 2024) and transmural and intramural helical organizations. With 7 T MRI, Nishitani et al. (2020) have found and visualized the intramural and transmural helix angle organization from 8 WGA/42 d.p.f. The functional development of cardiac activity could be monitored non‐invasively by fetal electrocardiography (Strasburger et al., 2022) and modelled (Pervolaraki et al., 2014) using the same methodology as developed for the adult heart (Panfilov & Holden, 1997).

The fetal growth of brain structures and fibre tracts can be studied *ex vivo* and in utero by MRI, and timelines for anatomical structures established (Thomason, 2020). The rapid growth in cells, dendrites, dendritic spines and axon branches from 26 to 32 WGA produces an excess of connections that can be edited or remodelled. Resting state functional connectivity mapping – the activity in the connectome – shows that large scale network and patterned activity begin prenatally, before sensory systems are formed, never mind operational, and peak around 25 w.p.f. (Jakab et al., 2014). Monitoring the pattern of movements can be used as an index of neurological maturation, and can be monitored by ultrasound. Evoked and spontaneous fetal brain activity can be studied by in utero magnetoencephalography (Lowery et al., 2006).

### 5.2 Placentation

The luminal epithelium of the endometrium grows over the implantation site, embedding the embryo and placenta within the endometrium of the uterine wall. Early villous formation starts 12–18 d.p.f. and lasts until 28 d.p.f. Throughout the rest of gestation all gas and metabolite exchange of the embryo and fetus is through the syncytiotrophoblast layer, which also produces hCG (Greenbaum et al., 2023; Ruane et al., 2022).

The endometrium is transformed by irreversible differentiation of myometrial into decidual stromal cells, characterized by downregulation of pro‐inflammatory response genes and upregulation of cell proliferation genes (Ng et al., 2020). Decidualization prevents maternal rejection of the invading embryonic cells, and at ∼8 WGA allows cytotrophoblast cells to invade and remodel the maternal myometrial spiral arteries, reducing their resistance by removing smooth muscle cells, and replacing their epithelial lining with fetal derived epithelium. The haemochorial placentae of mice and men (Georgiades et al., 2002; Siriwardena & Boroviak, 2022) provide the trophoblast with direct contact with maternal blood, and studies on transgenic mouse signalling mechanisms during implantation offer possible mechanisms for complications of human pregnancy, which later manifest as miscarriage, pre‐eclampsia and premature birth (Cha et al., 2012).

The placental volume increases slowly from implantation until 8 WGA when rapid growth starts, with the spiral arteries remodelled and highly dilated. Placental volume calculated from in vivo imaging increases 10‐fold from 9 WGA to full term birth (Abduljalil et al., 2012). The establishment of a functional placenta and embryo cardiovascular system allows rapid growth and development of the embryo, all underpinned by angiogenesis.

#### 5.2.1 µ‐Gravity

During 2–8 w.p.f. the embryo size increases more than 100‐fold, and an effective embryo–placental circulation is not established until ∼10 w.p.f., when maternal blood from decidual spiral arteries begins to penetrate the placental intervillous space. The increased supply of O_2_ and nutrients to the fetus enables the rapid growth in fetal volume from 10 WGA. The rapid growth moves towards space scales where hydrostatic effects of gravity on Earth influence bulk fluid movements between extracellular spaces, but these are regional in the mother, and expected to have little effect on the free floating embryo, or on the diffusive and oncotic flows across the embryo–maternal boundary.

#### 5.2.2 GCRs

The embryo offers an increasingly large target for interaction with GCRs, and in tissues and organs that are rapidly proliferating, an increasingly sensitive target. The effects of ionization induced damage would be an increased risk of malformations, which may be eliminated by early or late miscarriage, or later manifest in stillbirth, or in intrauterine growth restriction. The nuclear and cellular effects of GCR interactions may be lost, by repair or by cell death, or tissue or organ level effects may only emerge later. In the adult, radiation quality factors are associated with different organs. For the developing embryo and fetus, a measure of tissue sensitivity to radiation dose/unit mass would need to relate to developmental or birthing outcome, rather than risk of cancer; and would be expected to be higher when cell division rates are high and during periods of cell differentiation. All GCRs that reach the embryo and fetus have passed though the uterine wall, and effects following endometrial or myometrial inflammation produced by GCRs are likely, especially when the embryo and placenta offer 5–50 cm scale targets

## 6 FETAL GROWTH AND DEVELOPMENT: *P*_7_, *P*_8_, SPACE SCALE CENTIMETRES TO TENS OF CENTIMETRES

**FIGURE 4:**
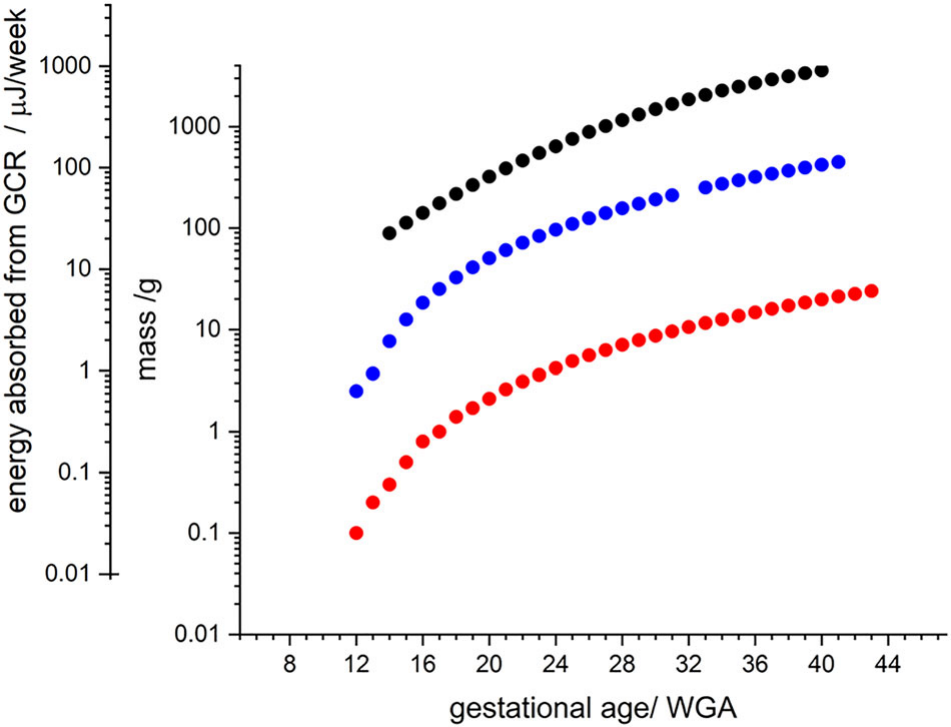
Growth of fetus, fetal heart and brain during prenatal development. Data from supplemental digital content of Abduljalil et al. (2019). Each fetal brain (blue) and heart (red) weight data point is the mean of a handful from postmortem cases. The fetal weight is estimated from volumes obtained in vivo by ultrasound. The energy absorbed is proportional to the mass (i.e. organs are assumed to be spherical), and the estimated energy absorbed/week during exposure to interplanetary GCRs is based on the ∼300 µGy day^−1^ dose rate of Zeitlin et al. (2013).

From 6 w.p.f. to full term, the fetus increases in mass from 10 g to ∼3–4 kg and in linear extent from 15 mm to 50 cm: repeated cell divisions increase the estimated number of cells from 1.3 × 10^9^ to 1.25 × 10^12^ diploid cells at birth (Osgood, 1955). This increase in mass or volume increases the target volume for interaction with GCRs (see Figure 4, where the average mass of fetal heart, brain and whole fetus is plotted on a logarithmic scale against gestational age). Since the mass of the brain is >10 times that of the heart, one would expect neuro‐cognitive effects to be commoner than cardiac effects of GCRs. The growth is by increase in number of cells and increase in cell size. During fetal growth cell divisions, increase in cell volume, differentiation, migration and cell death all occur at rates that vary with gestational age and that differ between tissues, and so the sensitivity to GCRs is expected to vary between tissues and with gestational age, and for any tissue be highest when there is differentiation and high growth rates. In adults wound healing is disturbed and slowed in space flight, partly via µ‐gravity actions on fibroblasts and organized collagen deposition, and partly via GCR actions on inflammatory processes (Babocs et al., 2025), and similar actions may disturb fetal growth.

Clinical observations of thalidomide induced embryopathy showed symmetrical upper limb malformations, which could be produced by a single dose, with a body half‐life of 8–12 h, within the window 20–36 d.p.f. when in the limb bud there is extensive cell movement and proliferation supported by angiogenesis. Earlier exposures before this vulnerable window result in miscarriages, later exposure in brain damage. Current theories for these time‐sensitive effects include thalidomide actions on angiogenesis, where new vessels that have not yet developed a vascular smooth muscle layer are vulnerable to thalidomide's actions, perhaps via ROS (Vargesson, 2015). However, tissues during early organogenesis in the first trimester seem more vulnerable to perturbations than later in gestation. A reasonable assumption is that the remodelling of fetal growth is similar to the remodelling of adult maintenance and growth, and that GCR effects will be mostly long term, and depend on the absorbed energy, or volume of tissue exposed.

How molecular processes inside and between cells lead to the emergence of gross morphogenesis cannot be directly studied in humans, but has been widely studied in mouse models. In mice Sonic Hedgehog signalling simulates polarization of morphogen gradients in in the limb bud and the neural tube, which are organizing centres (Briscoe & Thérond, 2013), and the hedgehog protein morphogens are transported by diffusive and cytomeme transport (Hall et al., 2024). Transcription factors that are promiscuously distributed throughout the embryo and interact with local transcriptional regulators can produce local patterning (Losa et al., 2023).

### 6.1 µGravity

The rapid growth from millimetre to centimetre length scales enables hydrostatic effects of gravity to become non‐negligible, and adult cardiovascular adaptations to µ‐gravity are qualitatively similar to those of pregnancy. Embryonic development in utero on Earth is in a neutral buoyancy environment that provides a plausible simulation of µ‐gravity, and fetal growth should not be different within the µ‐gravity of deep space.

### 6.2 GCRs

Tissues that are growing rapidly (cell division, migration and differentiation) may be more vulnerable to GCR tracks by intracellular interactions with their cell nuclei and mitosis producing long‐term genomic effects or local cell death. Ionizing interactions with membranes and cytoplasm could trigger inflammatory responses via ROS. There could also be indirect effects by actions on local angiogenesis. If fluctuations in GCR fluence are stationary throughout gestation, the probability of interactions of a cell with a GCR track will also be stationary, while the probability of interactions with a tissue or organ will scale with its size.

The nervous system is most sensitive to radiation between 8 and 15 WGA and relatively insensitive after 25 WGA: this roughly correlates with growth rate in neurone and neuroglial numbers (De Santis et al., 2007). Very specific, time sensitive effects, analogous to the time sensitivity of thalidomide, could be produced by GCR interactions, say 26–34 WGA, the critical period for fetal kidney growth, when the rate of kidney growth is highest, perhaps locked to local angiogenesis. However, tissues during early organogenesis in the first trimester seem more vulnerable to perturbations than later in gestation. A reasonable assumption is that any remodelling of fetal growth is similar to the remodelling of adult maintenance and growth, and that GCR effects will be mostly long term, and depend on the absorbed energy or volume of tissue exposed.

In Earth‐based controls of the effects of radiation on blastocyst development, radiation exposed blastocysts that were transferred into pseudo‐pregnant mice developed into live births (Lei et al., 2020). Radiation exposure of 2 mGy during blastogenesis reduced the incidence of live births by ∼75% in mice: this would scale the product (*P*_4_ × *P*_5_ × *P*_6_ × *P*_7_ × *P*_8_****** × *P*_9_) by 0.25.

## 7 MATERNAL ADAPTATIONS TO PREGNANCY

The physiological responses of adults during space flight, in free falling LEO and after return to Earth have been documented for over 500 individuals, with flight durations from a few to over 400 days. They are highly variable, even with similar flight durations, and include changes of 1–2%/month in bone resorption; up to 50% muscle atrophy, with increased fatigability and decreased force, speed and ability to regenerate (Lee et al., 2022); blood volume redistribution, with up to 50% increases in cardiac output and a decrease in mean arterial pressure and a lower systemic vascular resistance (Norsk, 2020); changes in cardiac function and structure (Sy et al., 2023); changes in vestibular reflexes (Clément et al., 2019), changes in immune system (Buchheim et al., 2019); structural changes in brain (Doroshin et al., 2022) and neuro‐cognitive performance (Mhatre et al., 2022); and changes in the microbiome (Kirkpatrick et al., 2020). The skeleto‐muscular changes can be limited by exercise regimes. During a period of convalescence these changes are all reversed on return to Earth. Many of these changes can be explained in terms the biophysics of muscular inactivity and lack of mechanical loading, and of centimetre to metre scale lack of gravitational hydrostatic pressure gradients causing regional redistribution of fluids, but are executed by intracellular and intercellular processes.

Maternal adaptions to pregnancy on Earth meet the increasing needs of the growing embryo and then of the fetus and placenta. Maternal blood volume increases from 6–8 WGA to ∼34 WGA when it plateaus at ∼150% pre‐pregnant plasma volume with an ∼40% increase in cardiac output. Changes in autonomic activity to arterioles (inferred from reduced heart rate variability and increased heart rate) limit any consequent rise in blood pressure. Oestrogens and NO levels contribute towards the systemic vasodilatation (Collins et al., 2024; Kuate Defo & Daskalopoulou, 2023). These maternal systemic cardiovascular responses to pregnancy are in the same direction as those seen to µ‐gravity in LEO, and presumably cardiovascular responses to pregnancy and µ‐gravity could be synergistic but would summate less than linearly.

### 7.1 µ‐Gravity

There have been no human pregnancies in space; on Earth the embryo and fetus float freely in a pool of amniotic fluid, and are subject to irregular maternal postural movements, both of which are qualitatively similar to simulations of µ‐gravity within a neutral buoyancy training tank or a clinostat. Fetal systemic responses to µ‐gravity would probably be secondary to any maternal changes in uterine arterial flow (Osol & Mandals, 2009; Moore et al., 2022; Sferruzzi‐Perri et al., 2023), which is increased 20‐fold during pregnancy and maternally regulated.

### 7.2 GCRs

The known effects of radiation in non‐pregnant women in LEO, where the bulk of solar particle radiation and GCRs have been reduced by the magnetosphere, may be considered as curiosities (phosphenes); minor (increased likelihood of cataracts) resulting from ROS via secondary ionization; or of significance: the increased risk of cancer and reduced life‐expectancy produced by DNA damage. Pregnancy‐specific effects would follow from the increased uterine size or be secondary to changed endocrine interactions with the embryo and fetus that result from embryo or fetal damage.

## 8 MYOMETRIAL PROGRESSION TOWARDS PARTURITION: *P*_8_, *P*_9_

In the non‐pregnant woman there are menstrual cycle related myometrial contractions, and travelling waves of excitation possibly producing peristalsis. During gestation the uterine mass and volume capacity increase from 70 g/10 mL at the time of implantation to ∼1 kg/5 L at full term, and myometrial electrical and mechanical activity is inhibited. The mechanisms of the initiation and emergence of the uterine‐wide contractions of labour are not clearly defined (Aslanidi et al., 2011; Taggart et al., 2007; Sheldon et al., 2014), but involve both non‐linear stochastic synchronization and travelling wave phenomena (Holden, 1976; 1986; Holden et al., 1991), mediated by cell and tissue electrophysiological excitation and mechanoelectrical feedback.

Preterm birth (before 37 WGA) rates are about 8% in the UK, and the earlier the birth the greater the risk of morbidity, which can be life‐long and last through a long life, and of neonatal mortality. The triggers for premature labour include infection and inflammatory responses, the full term labour myometrial transcriptome shows increased activity in pathways identified as inflammatory (Mittal et al., 2010), and labour has been characterized as an inflammatory event (Leimert et al., 2021). Premature labour contractions are often ineffective, but can lead to a drawn‐out delivery.

As the uterine myometrium expands during gestation it remains quiescent, with uterine smooth muscle cell (USMC) membrane potential hyperpolarized due to upregulation of K^+^‐selective conductances (Greenwood & Tribe, 2014; Wray & Arrowsmith, 2021) and downregulation of connexin‐43 (Chow & Lye, 1994). There is a fall in K^+^ channel expression, and increase in gap junctional expression, before full term birth, and before premature birth (Balducci et al., 1993). The human USMC membrane potential depolarizes from ∼−75 mV at 28 WGA to −45 mV at 40 WGA (Parkington et al., 1999), and within this period there is a 95% survival of premature births in a hospital environment. In premature labour these changes are often associated with infection and triggered inflammatory responses, mostly mediated via ROS (Menon, 2022).

Effective labour leading to birth requires engagement of the head, contractions driven by oxytocin and a ripened cervix. Plasma basal oxytocin concentration increases gradually during pregnancy, and transitioning from late pregnancy to early birth there are short pulses of oxytocin that increases in amplitude, duration and rate (Uvnäs‐Moberg, 2024).

### 8.1 µ‐Gravity

During much of gestation the fetus is floating within amniotic fluid, and is in a neutral approximation to free fall. Traditional birthing methods on Earth – walking, squatting, birthing chairs – exploit inbuilt gravity, and giving birth in a vertical position requires less interventions and pain relief (Desseauve et al., 2017). Horizontal birthing on a bed is more convenient for obstetricians, and floating in water birth is an attractive alternative that has little impact on outcomes (Cluett et al., 2018). Birth in µ‐gravity would require some confining tethering and containment of fluids lost. Blood, sweat and tears are not the only fluids voided during birth: as well as 500 mL blood, there will be about 800 mL of amniotic fluid, and incontinence. It is a messy business even on Earth where gravity keeps stuff together.

### 8.2 GCRs

The ∼1000‐fold increase in volume and 20‐fold growth in myometrial mass due to USMC hyperplasia and hypertrophy during gestation (Ono et al., 2015) will increase the rate of interaction between the background GCRs and myometrial cells. GCR nuclear interactions within USMC nuclei could trigger later cancer, or trigger local cell death that would be masked by the USMC hyperplasia. As 98.5% of the UMSC volume is extra‐nuclear (Sweeney et al., 2014), GCR interaction will be mostly cytoplasmic, leading to increased ROS production, along centimetre long, >100 µm diameter fuzzy tubes of ionization through the myometrial tissue. The GCR‐produced ionization would increase ROS and mimic inflammation, and could lead to a clustered cell excitation or lowered excitation threshold, resulting in an increased rate of local activity, and in more frequent, longer and larger responses to maternal oxytocin, and perhaps trigger spontaneous contractions. This would enhance the likelihood of premature or early labour as myometrial volume increases, and gap junctional expression increases and K^+^ conductances begin to fall after 28 WGA.

### 8.3 Closed environment

The fluid losses of childbirth will need to be dealt with; otherwise the fluid would float around as globules or a fine mist, adhere to surfaces, and dry out providing a substrate for microorganisms and biofilms.

## 9 NEONATAL NURTURE AND DEVELOPMENT: *P*_10_

The spacecraft crew quarters will not have been designed on the assumption that a baby will be floating about in it, and will contain hazards that can be reduced by babyproofing, monitoring, carrying the baby around, bound or tethered, and confining it as in a playpen. The probability of a successful postnatal development in the confined µ‐gravity environment will be decreased by the increased likelihood of premature birth, congenital defects, developmental disorders and delays, and defects hidden at birth that emerge later after birth, and defects acquired after birth.

On Earth breast is best both because, unlike baby‐milk formula products, it is nutritionally ideal and does not require clean water; in spaceflight breast‐feeding has the added advantage of not requiring additional stores, in‐built availability and ready access. Breast feeding is the only feeding needed for the first 6 months, and should be continued during weaning. The percentage of mothers unable to breast feed is <5%, and a premature birth may need supplementary feeding while breast feeding is being established.

On ISS humidity is regulated to be 25–75%, partly to limit microbiome proliferation. The barrier functions of fetal skin are developed by 34 WGA; premature infants born before then have excessive transepidermal water loss and need a high humidity and temperature‐controlled incubator. Competent skin barrier functions take 2–4 weeks to develop in premature babies born before 34 WGA (Kalia et al., 1998).

Brain growth, by proliferation of neurons, is not complete till 18 months after birth, and the brain is not fully mature till well after adolescence, if then. Rapid brain growth continues during the first 20 postnatal weeks (Thomason, 2020). The development of the brain is initially by the same processes as for other tissues and organs, and is sensitive to inflammatory processes (Bennet et al., 2018) but shifts from primarily cell division, migration and growth of neurones and neuroglia in the fetus to editing inappropriate synaptic connections and cell death of neurons in the infant. This is concomitant with axonal extension in a very small proportion of neurons, and maturation of myelination in an even smaller fraction: of the ∼10^12^ neurones in the newborn brain ∼10^7^ have axons longer than a few millimetres.

A newborn has some reflexes that could be imagined as postural – the tonic neck reflex, the Moro/startle reflex of flinging out the limbs as if to find something to cling on to, and the grasping reflex of tightly clinging on. Vestibular‐ocular reflexes are present at birth, and motor behaviour develops from random joyful extensions through head‐lifting to rolling and crawling with timescale milestones that can be used to monitor neurophysiological maturation and development (Cainelli et al., 2025).

### 9.1 µ‐Gravity

Even when constrained to the floor by gravity, babies get around, and in µ‐gravity even the random movements of a newborn will produce rotation, tumbling and drifting: there will be a need for tethering. How microgravity will impact on the newborn's learning of motor control, from eye movements fixating on faces into progressive goal directed movements would be fascinating to follow. The lack of congruence between reafferent motor and vestibular sensory signals in µ‐gravity would be expected to prevent the development of vestibular reflexes. This could pose problems on any later transfer to planetary gravity.

### 9.2 GCRs

The volume of the neonatal brain offers a large target for interaction with GCRs. X‐ray and proton beam irradiation of the mouse brain has shown doses of >60 Gy are needed before histological damage, whereas behavioural radiobiology has demonstrated cognitive defects in adults at 1.5 Gy. At the neuronal scale, most of the cell material available for interaction with GCR ionization tracks is dendritic. Exposure to proton beams (0.1–1 Gy) produces quantitative ultrastructural changes in adult mouse cortical dendritic structure (number, length, branching) and spine density, with ‘immature’ long thin spines being most sensitive (Parihar et al., 2015). The structural changes probably reflect changes in synaptic plasticity or editing analogous to those in the neonatal brain. The effects of GCRs on synaptic editing in the fetal and neonatal mammalian brain are unknown but could be studied on Earth in the GCR simulator (Simonsen et al., 2020), and models of libraries of GCR tracks interacting with branching dendritic geometries have been developed (Alp et al., 2015).

### 9.3 Closed environment

A birth would be an engaging experience for the crew and strengthen their group bonds. Although breast feeding is fairly tidy, its aftereffects, from burps and sicking‐up to uncontrolled urination and defecation several times a day will have a negative impact on the habitat, and the comfort of other fellow travellers.

*P*_10_ will be reduced by the effects of GCRs, µ‐gravity and the closed environment, but as there is no measure for successful postnatal development, this is not simply quantifiable.

## 10 CONCLUSIONS

No mammals have been conceived, borne through gestation and born in the freefall of LEO, never mind in interplanetary space, but probabilities can be induced from current knowledge of mammalian morphogenesis and human embryo, fetal and neonatal development on Earth. Here the population probability distributions for late miscarriage, still birth and birth are accurately known but disparate, and depend on local circumstance. The probability estimates of early stages, from ejaculation *P*_0_ to gastrulation *P*_5_, vary, but it is clear that most of the overall ∼70% post‐fertilization pregnancy loss is in the first 2 w.p.f. For an embryo, the probability of its survival drops rapidly in early gestation; once a fetus is established its survival is fairly secure.

The principal physical hazards of prolonged interplanetary flight on physiology are the exposure to GCRs and µ‐gravity; these will be experienced by the adult crew as well as any developing embryo or fetus. Size matters in the effects of both gravity and GCRs, and embryo and fetal growth offers an increasing target for interactions with GCRs.

The effects of long‐term free‐fall of LEO on human physiology are well documented – and mostly hydrostatic or musclo‐skeletal atrophy through disuse – and are reversed on return to Earth. No systemic effects of µ‐gravity on the embryo are expected – it is too small. Systemic effects of µ‐gravity on the fetus, free floating in the neutral buoyancy of amniotic fluid for most of gestation, are expected to be negligible, but there may be indirect effects resulting from changes in maternal blood flow and hydrostatic pressures. In the adult, cell division carries on much as usual in LEO, and so major direct µ‐gravity effects on cell division, differentiation and migration during embryogenesis are not expected. At the intracellular space scale behaviours are determined by intermolecular forces and charge. However, changes in gene expression in pathways associated with mitosis, apoptosis, differentiation and proliferation have been reported in cultured human stem cells and iPSC organoids (Huang et al., 2025; Marotta et al., 2024) in LEO and ascribed to µ‐gravity. Transcriptomic changes have been reported between before and after spaceflight (Overbey et al., 2024). If these are direct cellular responses, rather than cellular responses to systemic stress, an intracellular gravity sensor, perhaps spatially extended cytoskeletal structures (microtubules or filaments) is needed.

There is no direct experimental evidence of the effects of GCRs on cells and tissues, but GCR simulators are now available. The anticipated effects of GCRs will be on the cell nucleus, producing mutations, and on the cytoplasm, with ionization increasing ROS. The linear tracks produced by GCRs through the tissue are expected to have major effects at the cell and tissue levels, especially when they are more vulnerable to perturbation, during mitoses in cells and angiogenesis in organs. However, the small size of the embryo and fetus, and the functional completion of most organs by mid‐gestation means there is less tissue to interact with GCRs at these vulnerable times. Interactions of GCRs with the myometrium will almost certainly increase the probability of premature birth and its associated morbidities. However most babies can survive birth at 30 WGA, and with a little help from their friends in neonatal intensive care, from 24 WGA.

Pregnancy in interplanetary space need not be a disaster that must be avoided – it may well lead to a completely successful childbirth, and should be manageable. The possible outcomes are the same as on Earth, but the probabilities of successful outcomes of the different stages will be reduced, and the probability of a preterm delivery increased. There are two major questions: will implantation, gastrulation and organogenesis proceed sufficiently – if not, the pregnancy will be lost perhaps before the mother becomes aware of it; and will GCRs initiate uterine excitation leading to an early preterm delivery. The mechanisms of early pregnancy loss and of early preterm labour are poorly understood, of current clinical interest here and now, and need further basic biomedical and clinical investigations.

If a long‐term goal of establishing permanent settlements on other planets is serious, reproduction needs to be considered, and pregnancy not automatically avoided, but encompassed in plans as an unscheduled but permissible outcome. Given the demonstrated success of family groups in the spread of humanity over Earth, the possibility of a pregnancy could be positively facilitated by favouring compatible members in the crew selection process, making it a family affair.

## AUTHOR CONTRIBUTIONS

Sole author.

## CONFLICT OF INTEREST

None declared.

## FUNDING INFORMATION

None.

## ESTIMATION OF PROBABILITIES

### *P*_0_: probability of an intravaginal ejaculatory event

Sperm are continually produced post‐puberty at about a hundred million/day, and accumulated until they are voluntarily ejaculated or involuntarily emitted within ∼50 days, before they are resorbed (Amann & Howards, 1980; Misell et al., 2006). A typical ejaculate would contain 10^8^ sperm, of varying age and functionality (Levine et al., 2017).

The timing of impregnation can be controlled in farm and laboratory animals, but in ordinary domestic circumstances people choose their own times, and self‐reported schedules are unreliable. Some couples try to lock sexual activity to a phase of the menstrual cycle, to minimize or maximize the likelihood of conception. While there is often a habitual periodicity in sexual activity within individual pairs, there is large variability in the frequency of intravaginal ejaculatory events within paired couples in a population. Interplanetary travel is an extraordinary circumstance, so the probability of ejaculation within the 5 days before and including ovulation is effectively binary, either it happens, or it doesn't. To have a fixed starting point that maintains the possibility of a pregnancy *P*_0_ is set to 1: any assumptions about the likelihood and phase of menstrual cycle of any extra‐terrestrial sex would simply arbitrarily scale this and all downstream outcome probabilities.

### *P*_1_: Probability of ovulation

In the absence of a chemical contraceptive regime, ovulation occurs more or less regularly, and in a population has a cycle length of 28.1 ± 3.9 days (Chiazze et al., 1968). Some women have regular, some irregular, cycles, but a randomly selected, healthy, pre‐menopausal and non‐pregnant woman would have an external predictive probability of ∼0.03–0.04 of ovulating on any day. An individual would know her regularity and time since last period and her predictive probability would be ∼0.8, based on the failure rates of rhythm methods of birth control.

### *P*_2_: Probability of fertilization

Taking a 28‐day cycle length and day 1 as the start of the menstrual flow, ovulation occurs around day 14. Sperm can hang around for about 5 days and the ovum is viable for a day, and for fertilization to occur intravaginal deposition of sperm needs to occur within in the potentially fertile period of days 9–14, within which there is only 1 day when the ovum is available and fit to be fertilized. Ovulation and semen deposition in the appropriate time frame are necessary but not sufficient conditions for fertilization to occur.

Meiosis for oocyte formation in the mother began at about 9 WGA and continued until her birth, with nuclear maturation arrested at prophase 1. Meiosis resumes post‐puberty, when during each menstrual cycle the oocyte of the dominant follicle matures and is arrested into metaphase of meiosis 2 as a ∼100 µm diameter spherical ovum (Telfer et al., 2023).

The oocyte that is available for later fertilization is decades old, and this maternal age of the oocyte reduces its ovum's fertilizability (Zielinska et al., 2019) and the aging of the adult it develops into after fertilization and birth. (Cohen et al., 2022). Post‐ovulatory ageing, waiting for sperm to arrive, increases the risk of embryo failure (Wilcox et al., 1998).

The probability *P*_2_ of fertilization occurring in vivo when sperm delivery and ovulation are within ±24 h is difficult to estimate directly. The conception rate in a population of young women aiming to become pregnant is about 40%/cycle over the first year (Wang et al., 2003). The probability of a clinically identified pregnancy occurring in a given menstrual cycle is about 0.3–0.4, but this low fecundity is not just due to failure to fertilize, but is also due to early pregnancy loss after fertilization and before gastrulation and implantation (Macklon et al., 2002).

Lavage of surgically removed uterus and fallopian tubes in women who had demonstrated fertility has been used to retrieve embryos (Hertig et al., 1959), and the number retrieved within 24 h of documented sex and ovulation used to estimate *P*_2_ as 0.93. This high value may be biased and have a low precision (Jarvis, 2016). Pre‐embryo recovery after artificial insemination has led to estimates of *P*_2_ as 0.50 (Buster et al., 1985) and from natural cycle IVF as 0.75 (Formigli et al., 1990). These estimates are fractions, each based on different assumptions, techniques and populations, and could be set to near zero by use of contraceptives.

### *P*_3_: Probability of competent blastocyst

After fertilization, the 100 µm diameter zygote is passively moved towards and into the uterine cavity, while undergoing repeated cleavages to produce a pre‐implantation blastocyst (Zamboni et al., 1966). After fertilization the zygote and pre‐implantation embryo from ART can be visualized and studied in vitro (Mio et al., 2024). These in vitro studies allow mechanisms to be evaluated, but their success rates may reflect the technology rather than the in vivo probabilities, and assume that the recovered ova and conditions are fully competent for their fertilization and development. The single‐celled zygote begins a cascade of *cleavages*, into pairs of blastomeres of similar volume, that form the 100 µm diameter morula and lead to the blastocyst. Mitosis and cleavage are executed by intracellular actin filament interactions with myosin attached to internal and surface membranes, redistributing the cell contents.

Fertilization by IVF or intracytoplasmic sperm injection gives about 50% formation of blastocysts (Van Landuyt et al., 2005). in vivo, with the developing blastocyst bathed in uterine luminal fluid rather than a saline, the probability *P*_3_ of competent blastocyst formation given fertilization 5 days earlier can be estimated at 0.6–0.9 (Jarvis., 2016). *P*_3_ = 0.6 is used in Figure 2 as embryo survival is still robust.

### *P*_4_: Probability of implantation

Implantation rates of IVF blastocyst transfers are low at 10%–40%, and explain the use in ART of multiple transfers of multiple embryos, with the concomitant likelihood of multiple births, and need for pre‐transfer blastocyst screening and selection. Natural in vivo implantation rates are estimated to be higher, 60–90% (Jarvis, 2020), than for IVF, giving, *P*_4_ ≈ 0.6–0.9.

The immunoassay detection of an increase in hCG in maternal blood and urine samples 6–8 days after fertilization, around the time of implantation, can provide the first clinical identification of pregnancy. Taking [hCG] > 0.025 ng/mL on three consecutive days as indicative of pregnancy, 20% of these early chemical pregnancies failed to advance into clinically recognized pregnancies (Wilcox et al., 1988), and this early pregnancy loss could be considered as failed implantation or of decidualizaton, giving *P*_4_ ∼0.8. *P*_4_ = 0.6 is used in Figure 2 as embryo survival is still robust. Most conceptions terminate in clinically unrecognized early pregnancy loss, or in spontaneous early abortions.

### *P*_5_: Probability of gastrulation

During gastrulation, the vertebrate infrastructure of endo‐, ecto‐ and mesoderm germ layers emerges from the single endothelial layer of the blastocyst, symmetry breaking and elongation form the main body axes, and morphogenetic movements lead to the three‐dimensional structure of the gastrula.

Gastrulation occurs between 14 and 21 d.p.f., after the 14 d.p.f limit on keeping human embryos alive in vitro, and so experiments on human embryos are lacking after 14 d.p.f., or after the point when the embryo exhibits a primitive streak. Preimplantation gastrulating human embryos occasionally become clinically available, and spatially resolved single cell transcriptomic profiles of gastrulating human embryos have been obtained (Molè et al., 2021; Tyser et al., 2021; Xu et al., 2023) and primordial germ cells, red blood cells, and various mesodermal and endodermal cell types identified.

In some jurisdictions the 14 d.p.f., time limit has been legally side‐stepped by constructing models of human embryogenesis from human stem cells/induced pluripotent stem cells (de Graeff et al., 2023), where the in vitro ‘gastruloid’ or ‘embryon’ (Hamazaki et al., 2024; Liu et al., 2023; Oldak et al., 2023; Weatherbee et al., 2023) is genetically human, morphologically mimics a human embryo, but is physiologically, developmentally and epigenetically whatever. Gastrulation in gastruloids was identified by the presence of markers for epiblast‐ and hypoblast‐like cells, and of yolk sac‐ and amniotic‐like cavities. The success rates of gastruloid gastrulation may reflect the criteria and technology rather than the success rates of in vivo gastrula gastrulation, and were about 75%.

Errors in gastrulation can be incompatible with development of a functional fetus, and are believed to be a main source for the 10–25% of clinically identified pregnancies that end in miscarriage (Zinaman et al., 1996), giving *P*_5_ = 0.75–0.9.

### *P*_6_: Probability of successful placentation

The placenta is the first embryo organ to develop and function, and successful placentation starts with implantation as the trophectoderm attaches to the endometrial epithelial surface to form a syncytium at 6–7 d.p.f. The resultant trophoblast is highly invasive, syncytial cells invade the endometrial decidua, and by 14 d.p.f. the blastocyst is embedded in the decidua. The embedded blastocyst is surrounded by a syncytiotrophoblast surface layer (Gauster et al., 2022; Turco & Moffett, 2019), the interface for all gas and metabolite exchange between the embryo and maternal tissues.

Abnormal development of the placenta is a major contributing factor to many of the complications of pregnancy that contribute to fetal morbidity and death. Failure to form a functional placenta at the millimetre space scale merges with implantation failure in contributing to the probability of pre‐clinical pregnancy loss. Post‐implantation abnormal development of the placenta at the centimetre to tens of centimetres scale will contribute to the probability of early and late clinical pregnancy loss, and maternal morbidity and mortality (Brosens et al., 2011).

The probability of failure of the trophoblast to invade after implantation is essentially zero; it is highly invasive, and in 1.1% of all pregnancies invades inappropriate ectopic sites. However, the placenta and placental blood vessels may later develop over the cervix, leading to fetal/embryo loss and contributing to maternal deaths. These inappropriate location placentation failure probabilities lead to *P*_6_ = 0.98. Three‐quarters of the pregnancies that are lost after ∼22 WGA (stlllbirths) have been ascribed to physiological ripples or aftershocks resulting from inadequacies in earlier placentation (Cha et al., 2012). The physiological consequences of errors in implantation, gastrulation, decidualization and placentation may only emerge much later in development (Silver, 2015), and contribute to 75% of stillbirths. Small quantitative problems early on in gestation can cascade through development, leading into quantitative or qualitative developments that are non‐viable later on.

Taking clinical pregnancy loss as about 1% after 22 WGA (French, Bierman, 1962) the probability of placentation success *P*_6_ = 0.975–0.98.

### *P*_7_: Probability of successful organogenesis

By 21 d.p.f. organogenesis is beginning within a 1.5–2 mm CS 20 embryo, and as the feto‐placental circulation becomes functional, rapid growth and development of the embryo occurs. The principal organs are discernible by 6 w.p.f. within a recognizable mammalian body plan, now the fetus, and organogenesis continues throughout fetal development. A premature baby born at 32 WGA has a 95% chance of survival, and so organogenesis, and its integration, is physiologically functional. Before 24 WGA, viability, even in neonatal intensive care, is negligible.

Failures in organogenesis can be inferred from pregnancy loss statistics, or from the incidence of congenital malformations. Currently accepted pregnancy loss figures as a percentage of clinically identified pregnancies are 10–20% for first trimester, and 1–2% for second trimester (late) miscarriages, and 0.4% stillbirths in the third trimester. The incidence of stillbirth/10,000 births rises from ∼2 to ∼10 from 37 to 42 WGA (Rosenstein et al., 2012). Late miscarriages and stillbirths may be due to earlier failures in organogenesis that only later impact sufficiently on fetal physiology to be lethal, but 35% of stillbirths have no apparent morphological abnormality, and could be physiological, say due to a cardiac arrhythmia (Pervolaraki et al., 2013). If all late miscarriage and stillbirth are blamed on failures in organogenesis, this would give up to a 0.024 contribution to the probability of organogenesis failure.

Congenital birth defects are present in about 3% of live births, congenital heart disease in about 1% (Bouma & Mulder, 2017), and neural tube defects in 0.2% of live births (Kancherla, 2023), and about 20% of congenital defects are associated with genetic factors. Congenital birth disorders may be invisible and only manifest after birth, as failures to reach developmental milestones. If 80% of congenital defects were ascribed to failures in organogenesis, they would contribute up to 0.024 to the probability of organogenesis failure.

Where a cause or trigger has been identified it has a maximal impact during a developmental time window from 3 to 12 w.p.f. even if the effect is only apparent later. If failures in organogenesis during 3–12 w.p.f. were responsible for all early, late miscarriage, still birth and 80% of congenital birth defects *P*_7_ ≈ 0.952.

### *P*_8_: Probability of normal gross morphology

Successful organogenesis is not sufficient for development of a normal gross morphology; it needs also to be completed in the appropriate sequence and at the appropriate times. Perturbations in the timing, or increased sensitivity to perturbations at critical times, can lead to embryopathies that are macroscopically visible structural malformations that involve several tissues types in one or more body parts. Examples are limb deformations produced by exposure to thalidomide.

Of the ∼3% of births with congenital defects, few involve multiple parts, and specimens of such rarities have been historically collected into anatomical museums (Kosenko et al., 2022), and a study of teratology has informed developmental biology. They are sufficiently rare for *P*_8_ to be taken as 0.97.

### *P*_9_: Probability of successful birth

Human delivery is complicated: rotation and squeezing the fetal head and shoulders through the birth canal can be a long drawn out and difficult process that makes birth assistance obligatory (Mitteroecker & Fischer, 2024). Childbirth can be a dangerous process, for both the mother and the child, with in‐hospital maternal death rates of ∼0.013% and neonatal death rates 0.3% of live births in the UK. These include preterm births.

For labour to deliver, the contractions need to be effective and coincide with cervical ripening, a collapse in the visco‐elastic properties of the cervical ring, that allows the cervix to dilate. Dysfunctional parturition can result from ineffectual myometrial activity or ineffectual cervical ripening, both leading to poor fetal outcomes. Failure of the myometrium to contract effectively after birth causes obstetric haemorrhage, a major cause of maternal death. If a successful outcome of a full term birth is mother and baby both alive, *P*_9_ ≈ 0.997; if it is both alive and well *P*_9_ ≈ 0.96; both these probabilities would be reduced in preterm births.

### *P*_10_: Probability of successful postnatal development

Neonatal (within 28 days after birth) and infant (within one year) mortality rates/1000 births in the UK are 2.9 and 4, giving a probability of having any postnatal development of 0.996. This would be lower for a premature birth. A successful postnatal development (Buijtenijk et al., 2003) can be indicated by achieving milestones, and can be limited by disease. It is primarily determined by adequate and appropriate nutrition, social interactions and societal (economic and equality) rather than physiological factors (Weightman et al., 2012). Taking surviving to a year as an index of successful postnatal development, *P*_10 _= 0.996; any more ambitious indices would lower this.

The creq quarters will not have been designed on the assumption that a baby will be floating about in it, and will contain hazards that can be reduced by babyproofing the habitat, monitoring, carrying the baby around, bound or in a sling, and confining it in a playpen. The probability of a successful postnatal development is for a peculiar environment, but much the same as on Earth, but will be decreased by increased likelihood of premature birth, congenital defects, disorders and defects visible at birth, and cognitive or behavioural defects or delays.
